# Genetic partitioning of interleukin-6 signalling in mice dissociates Stat3 from Smad3-mediated lung fibrosis

**DOI:** 10.1002/emmm.201100604

**Published:** 2012-06-08

**Authors:** Robert J J O'Donoghue, Darryl A Knight, Carl D Richards, Cecilia M Prêle, Hui Ling Lau, Andrew G Jarnicki, Jessica Jones, Steven Bozinovski, Ross Vlahos, Stefan Thiem, Brent S McKenzie, Bo Wang, Philip Stumbles, Geoffrey J Laurent, Robin J McAnulty, Stefan Rose-John, Hong Jian Zhu, Gary P Anderson, Matthias R Ernst, Steven E Mutsaers

**Affiliations:** 1 Ludwig Institute for Cancer Research, Melbourne – Parkville BranchParkville, Victoria, Australia; 2 Centre for Asthma Allergy and Respiratory Research, School of Medicine and Pharmacology, University of Western AustraliaNedlands, Western Australia, Australia; 3 Lung Institute of Western AustraliaNedlands, Western Australia, Australia; 4 Departments of Pharmacology and Medicine, University of MelbourneMelbourne, Victoria, Australia; 5 UBC James Hogg Research Centre, Heart+Lung InstituteVancouver, BC, Canada; 6 Department of Pathology and Molecular Medicine, McMaster UniversityHamilton, Ontario, Canada; 7 CSL Ltd, Bio21 InstituteParkville, Victoria, Australia; 8 Department of Surgery, University of MelbourneMelbourne, Victoria, Australia; 9 Telethon Institute for Child Health Research, Centre for Child Health Research, University of Western AustraliaWestern Australia, Australia; 10 Centre for Respiratory Research, Rayne Institute, Royal Free and University College LondonLondon, UK; 11 Department of Biochemistry, Christian-Albrechts-Universität zu KielKiel, Germany; 12 PathWest Laboratory Medicine WANedlands, Western Australia, Australia

**Keywords:** interleukin 6, pulmonary fibrosis, Smad3, Stat3, transforming growth factor beta

## Abstract

Idiopathic pulmonary fibrosis (IPF) is a fatal disease that is unresponsive to current therapies and characterized by excessive collagen deposition and subsequent fibrosis. While inflammatory cytokines, including interleukin (IL)-6, are elevated in IPF, the molecular mechanisms that underlie this disease are incompletely understood, although the development of fibrosis is believed to depend on canonical transforming growth factor (TGF)-β signalling. We examined bleomycin-induced inflammation and fibrosis in mice carrying a mutation in the shared IL-6 family receptor gp130. Using genetic complementation, we directly correlate the extent of IL-6-mediated, excessive Stat3 activity with inflammatory infiltrates in the lung and the severity of fibrosis in corresponding *gp130*^*757F*^ mice. The extent of fibrosis was attenuated in B lymphocyte-deficient *gp130*^*757F*^;*µMT*^−/−^ compound mutant mice, but fibrosis still occurred in their *Smad3*^−/−^ counterparts consistent with the capacity of excessive Stat3 activity to induce *collagen 1α1* gene transcription independently of canonical TGF-β/Smad3 signalling. These findings are of therapeutic relevance, since we confirmed abundant STAT3 activation in fibrotic lungs from IPF patients and showed that genetic reduction of Stat3 protected mice from bleomycin-induced lung fibrosis.

## INTRODUCTION

Tissue fibrosis results from excessive and progressive scarring associated with destruction of normal tissue architecture and structure, and ultimately compromises organ function (Wilson & Wynn, 2009; Wynn, 2007). The clinical challenge of treating fibrotic diseases is exemplified by idiopathic pulmonary fibrosis (IPF), a heterogeneous disease unresponsive to therapy and fatal in outcome (Knight et al, 2003; Wilson & Wynn, 2009). Although the molecular mechanisms underlying initiation of IPF remain largely unknown, fibrosis is thought to arise from excessive tissue response to injury. Accordingly, effort has concentrated on the genetic dissection of steps that collectively govern normal wound healing processes and that enable re-epithelialization and extracellular matrix production to subside upon re-establishment of tissue homeostasis. Besides epithelial proliferation, differentiation and regeneration, these processes also involve stromal components, which are activated as part of the ensuing inflammatory response (Wilson & Wynn, 2009).

In IPF, epithelial injury is followed by pathologic fibrotic repair in distinct cellular foci within the lung parenchyma comprising proliferating fibroblasts and subepithelial myofibroblasts that are associated with excessive deposition of extracellular matrix proteins, including type I collagen (Cool et al, 2006; Maher et al, 2010; Moodley et al, 2003). Activation of these myofibroblasts correlates with increased levels of interleukin (IL)-1β, IL-4, IL-13, IL-17, and other inflammatory cytokines and is believed to be mediated by transforming growth factor (TGF)-β (Wilson & Wynn, 2009; Wilson et al, 2009). TGF-β and its canonical downstream signalling molecule Smad3 are central to the development and progression of fibrosis as elevated levels of TGF-β are sufficient to reproduce organ fibrosis in animal models, stimulate fibroblast differentiation and epithelial-to-mesenchymal transformation, and the observation that Smad3-deficiency confers resistance in mouse models of IPF (Bonniaud et al, 2004; Zhao et al, 2002).

During inflammation, stromal cells and those of the macrophage/monocyte lineage release inflammatory cytokines of the IL-6 family, which are thought to promote fibrosis through Erk1/2 signalling-associated fibroblast proliferation (Moodley et al, 2003) and the induction of a fibrotic response that is mediated by various TGF-β family of ligands (Ogata et al, 2006). Several members of the IL-6 cytokine family, which is characterized by the shared use of the common gp130 receptor subunit, have been implicated in pulmonary fibrosis. Transgenic overexpression of IL-11 or Oncostatin M (Osm) in mice, for instance, promotes lung scarring with striking histo-pathological similarities to that observed in human disease (Bamber et al, 1998; Kuhn et al, 2000). Meanwhile, overexpression of IL-6 is a common finding in bronchoalveolar lavage (BAL) fluid of IPF patients (Mozaffarian et al, 2008), where *IL6* gene polymorphisms segregate with disease severity (Pantelidis et al, 2001). Meanwhile, activation of the latent transcription factor Stat3, one of the signalling molecules engaged by gp130, has been proposed to affect fibrosis in skin and liver (Ghazizadeh et al, 2007; Ogata et al, 2006), albeit with contradicting outcomes (Mair et al, 2010). These observations therefore leave the mechanisms unresolved by which the wide-spread expression of gp130 on epithelial, stromal and hematopoietic cells, and the individual intracellular molecular components engaged by gp130 contribute to fibrosis and whether this response requires canonical TGF-β/Smad3 signalling (Knight et al, 2003).

In this study, we use gp130 mutant mice with either deregulated Stat1/3 or Erk1/2-signalling to assess susceptibility to bleomycin administration as a widely used model that recapitulates epithelial injury-induced lung fibrosis (Moeller et al, 2008). We found that ligand-dependent excessive Stat1/3 activation, either in the hematopoietic or stromal compartments of *gp130*^*757F*^ mice (Jenkins et al, 2005a), increased bleomycin-induced fibrosis in an IL-6 dependent manner, and that systemic ablation of *Il6* or impairment of gp130-mediated Stat3 activation, attenuated the fibrotic response. Importantly, gp130-mediated lung fibrosis occurred independently of canonical TGF-β signalling in Smad3-deficient mice, but required mature B lymphocytes and correlated with parenchymal accumulation of B-cell containing foci. With the prevalence of excessive Stat3 activation in lungs of IPF patients and the capability of therapeutically targeting components of the gp130 signalling cascade, our findings are likely to be of clinical relevance.

## RESULTS

### Excessive fibrotic response in mice with exaggerated IL-6-dependent Stat3 hyperactivation

**Figure 1:**
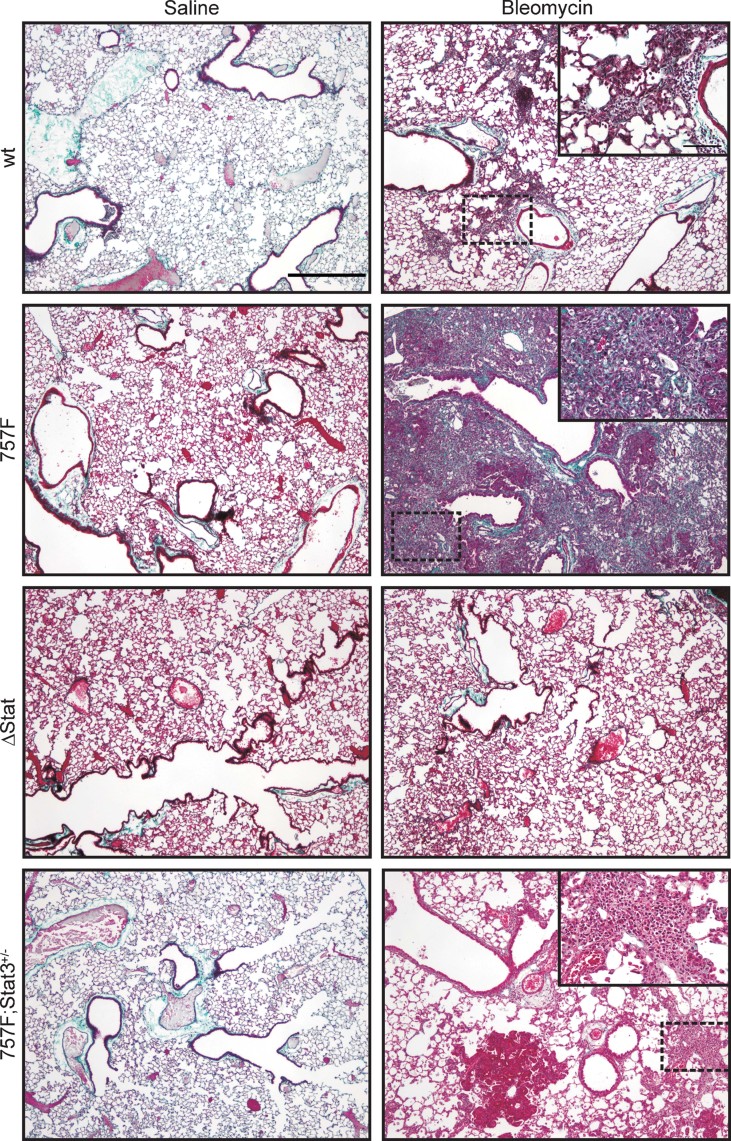
Gp130 cytokine family-mediated Stat3 signalling determines susceptibility to fibrosis Masson's trichrome stain of lungs from *gp130^wt^* (wt), *gp130^757F^* (757F), *gp130^ΔStat^* (ΔStat) or *gp130^757F^*;*Stat3*^+/−^ (757F;Stat3^+/−^) mice 30 days after saline or bleomycin treatment. Images are representative of three mice for each genotype. Scale bar = 500 µm (= 100 µm insets).

**Figure 2:**
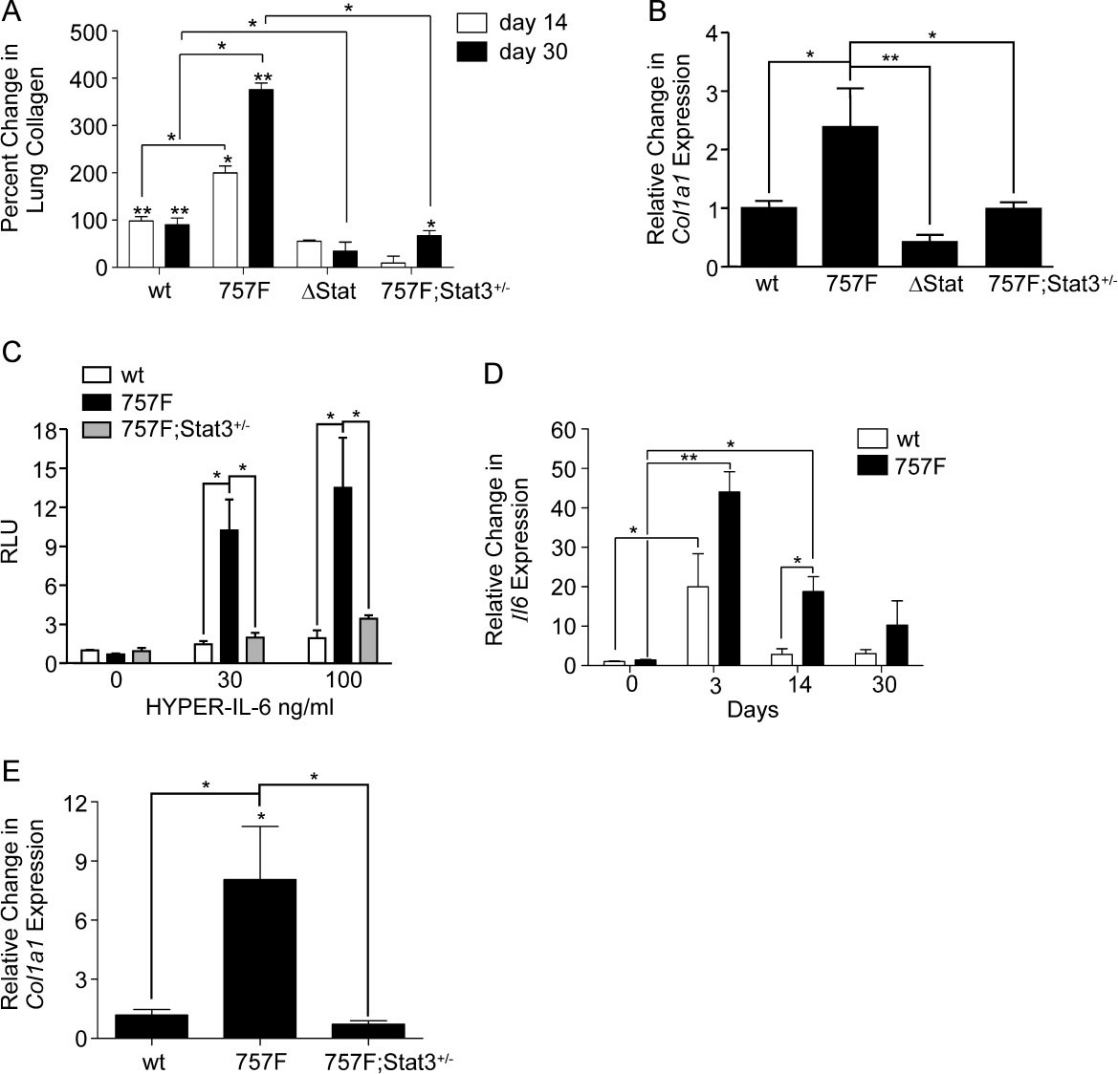
Gp130-mediated Stat3 signalling stimulates collagen accumulation and collagen transcription Percent change in collagen content in lung homogenates between saline and bleomycin-treated wild-type (wt), *gp130*^*757F*^ (757F), *gp130*^*ΔStat*^ (ΔStat) and *gp130*^*757F*^;*Stat3*^+/−^ (757F;Stat3^+/−^) mice 14 and 30 days after bleomycin challenge. Data were normalized to mean collagen content of syngeneic saline controls and expressed as percentage change. *n* ≥ 4 mice. The range of collagen between saline and bleomycin treated mice was 3.219–44.710 mg.qPCR analysis of *Col1a1* mRNA expression in lung homogenates 30 days after bleomycin treatment and normalized to *Gapdh* expression. *n* = 4 mice.HYPER-IL-6-dependent stimulation of *Col1a1-luc* reporter activity in transiently transfected embryonic fibroblasts. Data were normalized to Renilla luciferase activity and expressed as the relative change in relative luminescence units (RLU) compared to untreated syngeneic cells. *n* = 3 mice.qPCR analysis of *Il6* mRNA expression in lung homogenates from mice 3, 14 and 30 days after and before bleomycin challenge and control mice (0). *n* = 4 mice.qPCR analysis of *Col1a1* mRNA expression in lung homogenates following 2 weeks of trans-nasal HYPER-IL-6 delivery. *Col1a1* signals are expressed relative to the *Col1a1*/*18S* ratio of HYPER-IL-6 challenged wt mice. *n* = 4 mice. All data are expressed as mean ± SEM with *p < 0.05, **p < 0.01 using Bonferroni multiple comparisons test.

To mimic the development of human inflammation-associated lung fibrosis in mice, we trans-nasally administered bleomycin to gender-matched mice (8–12 weeks of age) harbouring gp130 mutations that bias intracellular signalling either towards the Stat1/3 or Erk1/2 signalling pathways in *gp130*^*757F*^ mice or *gp130*^*ΔStat*^ mice, respectively (Tebbutt et al, 2002) (Supporting Information Fig S1). Compared to wild-type (*gp130*^*wt*^) mice, bleomycin-challenged *gp130*^*757F*^ mice showed extensive changes to their lung architecture, including consolidation of airspaces, thickened alveolar septae, inflammation and epithelial dysplasia (Fig 1). In contrast, *gp130*^*ΔStat*^ mice were completely protected from pulmonary fibrosis. We corroborated these observations by measuring hydroxyproline content of lungs as an established marker of collagen deposition 14 and 30 days following bleomycin challenge, as well as by assessing *Col1a1* transcription in lungs of these mice (Fig 2A and B). We have previously shown that *gp130*^*ΔStat*^, *gp130*^*wt*^ and *gp130*^*757F*^ mice simultaneously comprise an allelic series for increasing gp130-mediated Stat1/3 signalling (attenuated in *gp130*^*ΔStat*^ and excessive in *gp130*^*757F*^ mice) and for Erk1/2 signalling (excessive in *gp130*^*ΔStat*^ and attenuated in *gp130*^*757F*^ mice) (Jenkins et al, 2005a; Tebbutt et al, 2002). This was confirmed by examining the abundance of transcriptionally active, tyrosine phosphorylated form of Stat3 (pStat3) in lung fibroblasts obtained from *gp130*^*757F*^, *gp130*^*wt*^ and *gp130*^*ΔStat*^ mice treated with IL-6 for 0–180 min (Supporting Information Fig S2A). The molecular rationale underpinning the reciprocal relationship between activation of the Stat1/3 and Erk1/2 signalling arises from the observation that the negative regulatory Socs3 protein is transcriptionally induced by Stat3 and requires binding to the phosphorylated tyrosine residue in position 757 in mouse (759 in human) gp130 (Ernst & Jenkins, 2004).

In order to dissect the contribution of the individual pathways engaged by gp130 to the fibrotic response, we systemically restricted the pool of Stat3 or Stat1 available for activation using compound *gp130*^*757F*^ mice (Ernst et al, 2008). Upon reduction of excessive Stat3 activation observed in compound *gp130*^*757F*^ mice to levels more comparable to those observed in *gp130*^*wt*^ mice, we detected a similar fibrotic response between *gp130*^*757F*^;*Stat3*^*+/−*^ and *gp130*^*wt*^ mice 30 days after challenge (Fig 1 and 2A-B). However, complete *Stat1* ablation in *gp130*^*757F*^;*Stat1*^−/−^ mice only provided partial protection from fibrosis (Supporting Information Fig S2B and C). These genetic observations imply that the enhanced fibrosis observed in *gp130*^*757F*^ mice mediated by increased Stat3 activation exceeds that mediated by genetic ablation of Stat1 expression (Walters et al, 2005) and suggest a direct relationship between the severity of the fibrotic response and the extent of gp130-mediated Stat3 signalling. Consistent with this, we detected excessive *Col1a1* luciferase (*Col1a1-luc*) reporter activity in *gp130*^*757F*^ embryonic fibroblasts in response to stimulation with the pan-gp130 designer cytokine HYPER-IL-6 (Fig 2C). Since HYPER-IL-6 activates gp130 independently of the ligand binding IL-6 receptor α-subunit (Fischer et al, 1997), this excludes the possibility that our observed results reflect potential differences in endogenous IL6 receptor expression between the different genotypes. We measured mRNA levels of the IL-6 family members *Il6*, *Il11* and *Osm* in the lungs of wt and *gp130*^*757F*^ mice 3 days after bleomycin challenge and found that in bleomycin-challenged lungs of *gp130*^*757F*^ mice, *Il6* mRNA remained selectively elevated 14 days later (Fig 2D and Supporting Information Fig 2D). Trans-nasal administration of HYPER-IL-6 promoted *Col1a1* gene transcription profoundly in lungs of *gp130*^*757F*^ mice relative to lungs of *gp130*^*757F*^;*Stat3*^+/−^ or *gp130*^*wt*^ mice (Fig 2E). The potential role of IL-6 family molecules directly promoting lung fibrosis was further supported by our observation that genetic ablation of *Il6* in *gp130*^*757F*^;*Il6*^−/−^ mice ameliorated the excessive fibrotic responses induced in bleomycin challenged *gp130*^*757F*^ mice (Supporting Information Fig S3A and B).

### Excessive fibrotic response in gp130^757F^ mice depends on mature B lymphocytes

**Figure 3:**
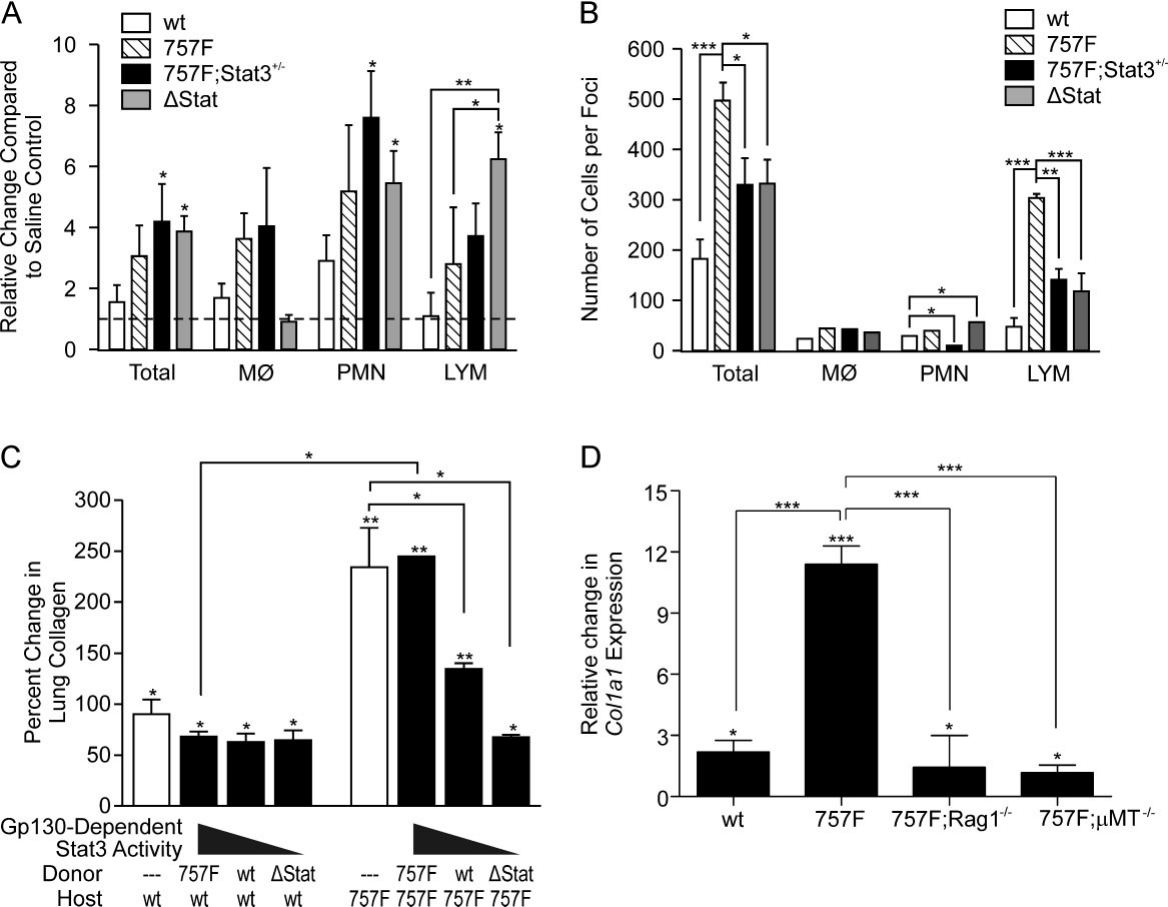
Increased lymphocytes are associated with inflammation and lung fibrosis Changes to cell numbers in BAL fluid of wild-type (wt), *gp130*^*757F*^ (757F), *gp130*^*ΔStat*^ (ΔStat) and *gp130*^*757F*^;*Stat3*^+/−^ (757F;Stat3^+/−^) mice 3 days after bleomycin administration relative to saline-treated mice of the same genotype. *n* ≥ 4 mice. MØ, macrophages; PMN, polymorphonuclear cells and LYM, lymphocytes.Distribution of inflammatory cells in foci within the lung parenchyma of mice 3 days after bleomycin treatment. Cell types were identified by histological appearance (PMN) or immunohistochemical staining for F4/80 (MØ), or CD3 and B220 (LYM). *n* ≥ 4 mice.Female wt or 757F mice (*n* > 3 per group) were reconstituted with bone marrow from 757F, wt or ΔStat mice and lungs were collected challenged 21 days after bleomycin or saline administration. Bars indicate percent change in collagen content in lung homogenates between saline and bleomycin treated mice. The range of collagen between saline and bleomycin treated mice was 7.089–31.658 mg. Empty bars show lung collagen levels in bleomycin challenged mice that have not undergone bone marrow transplant.qPCR analysis of *Col1a1* mRNA expression in lung homogenates 30 days after bleomycin challenge of wt, 757F, *gp130*^*757F*^;*Rag1*^−/−^ (757F;Rag1^−/−^) and *gp130*^*757F*^;*µMT*^−/−^ (757F;µMT^−/−^) mice. *Col1a1* signals were normalized to *18S* and expressed relative to saline-treated mice of the same genotype. *n* = 4 mice. All data are expressed as mean ± SEM with *p < 0.05, **p < 0.01 and ***p < 0.001 using Bonferroni multiple comparisons test.

Injury-dependent induction of cytokines primes the subsequent inflammatory response that precedes the development of pulmonary fibrosis (Wilson & Wynn, 2009). We therefore analysed BAL fluid from bleomycin-challenged mice and observed augmented cytokine accumulation, in particular of IL-1, IL-6, IL-13, and GCSF, in *gp130*^*757F*^ mice when compared to *gp130*^*wt*^, *gp130*^*757F*^;*Stat3*^*+/−*^, or *gp130*^*ΔStat*^ mice (Supporting Information Fig S4A and B). However, at the height of the inflammatory response 3 days after bleomycin administration (Moeller et al, 2008; Wilson & Wynn, 2009), there was no correlation between the number of inflammatory cells contained within BAL fluid of bleomycin-challenged mice (Fig 3A and Supporting Information Fig 4C) and extent of fibrosis 30 days later (Figs 1 and 2A and Supporting Information Fig 3A and B). Foci of inflammatory cells within the lung parenchyma were most prominent in *gp130*^*757F*^ mice compared to all other genotypes of mice consistent with the severe fibrosis observed in these mice (Fig 3B and Supporting Information Fig S5A). Collectively, these data suggest that attenuated Stat3 activation may enable egression of inflammatory cells into BAL fluid, while excessive Stat3 activity may promote their retention in the lung parenchyma.

Since bleomycin-induced lung fibrosis depends on the preceding inflammatory response (Moeller et al, 2008), we performed adoptive bone marrow transfer experiments to compare the contributions to disease between the hematopoietic and the parenchymal compartment. We excluded a major effect from the irradiation process, since the fibrotic response remained indistinguishable between non-irradiated and non-reconstituted bleomycin challenged *gp130*^*757F*^ or *gp130*^*wt*^ mice and their syngeneically reconstituted counterparts (Fig 3C). However, in reconstituted *gp130*^*757F*^ hosts, but not in *gp130*^*wt*^ hosts, we observed a gradual attenuation of bleomycin-induced hydroxyproline accumulation that correlated with the extent by which gp130-dependent Stat3 activation could occur in donor cells (*i.e.* excessive in *gp130*^*757F*^ and attenuated in *gp130*^*ΔStat*^ bone marrow cells). Furthermore, when reconstituted with bone marrow proficient for gp130-dependent Stat3 signalling (*i.e. gp130*^*757F*^ or *gp130*^*wt*^ cells), *gp130*^*757F*^ hosts were more susceptible to fibrosis than *gp130*^*wt*^ hosts. Collectively, these observations suggest that excessive gp130-mediated Stat3 signalling in the lung parenchyma of *gp130*^*757F*^ hosts promotes fibrosis, which is further amplified by excessive Stat3 signalling in bone marrow-derived cells.

The striking correlation between focal lymphocytic accumulation in the lung parenchyma of *gp130*^*757F*^ mice and the severity of their fibrotic response (Figs 2A and 3B) is consistent with a role for lymphocytes during development of bleomycin-induced lung fibrosis and their prominent abundance in fibrotic tissues of IPF patients with non-specific interstitial pneumonia (Keogh & Limper, 2005; Wilson & Wynn, 2009). Indeed, we observed a profound increase in B220-positive cells in bleomycin-challenged *gp130*^*757F*^ mice, which persisted for 30 days (Supporting Information Fig S5B and C). Furthermore, mature lymphocytes exacerbate fibrosis in *gp130*^*757F*^ mice, since fibrosis in T and B lymphocyte compound-deficient *gp130*^*757F*^;*Rag1*^−/−^ mice was reduced and comparable to disease in bleomycin challenged wild-type mice (Supporting Information Fig S5D). This observation was corroborated at the level of *Col1α1* gene transcription which was similar in wild-type and *gp130*^*757F*^;*Rag1*^−/−^ mice (Fig 3D). Moreover, we also observed reduced fibrosis and collagen transcription in *gp130*^*757F*^;*µMT*^−/−^ compound mutant mice deficient in mature B lymphocytes. Collectively, our findings suggest that bone marrow derived B lymphocytes promote bleomycin-induced fibrosis in susceptible *gp130*^*757F*^ hosts (Fig 3D and Supporting Information Fig S5D).

### TGF-β response is blunted in *gp130*^*757F*^ mice

The development of experimental fibrosis correlates with elevated TGF-β levels, which is thought to molecularly link the activity of inflammatory cytokines to the resulting fibrotic response (Bonniaud et al, 2005; Gauldie et al, 2007). This concept is supported by the observation that two distinct *Smad3* null mutations protect mice from bleomycin-induced lung fibrosis (Bonniaud et al, 2004; Zhao et al, 2002) as well as TGF-β-induced lung fibrosis (Bonniaud et al, 2004; Bonniaud et al, 2005). Using recombinant TGF-β1 as a reference, we detected elevated levels of TGF-β activity in serum from *gp130*^*757F*^ mice when assayed on NIH3T3 cells expressing the *pCAGA*_*12*_*-luc* reporter plasmid that records Smad3-dependent gene transcription (Supporting Information Fig S6A). However, when assaying for TGF-β-induced phosphorylation of Smad3 (pSmad3) we observed reduced abundance of this transcriptionally active form of Smad3 in lung fibroblasts from *gp130*^*757F*^ mice compared to those prepared from *gp130*^*wt*^, *gp130*^*ΔStat*^ or *gp130*^*757F*^;*Stat3*^+/−^ mice (Supporting Information Fig S6B). Similarly, the capacity of TGF-β to induce *Col1a1* gene expression was reduced in lung fibroblasts from *gp130*^*757F*^ mice when compared to those obtained from either *gp130*^*wt*^ or *gp130*^*757F*^;*Stat3*^+/−^ mice (Supporting Information Fig S6C), suggesting that excessive Stat3 activation decreases TGF-β-responsiveness of primary lung fibroblast from *gp130*^*757F*^ mice. Indeed, this observation is consistent with our previous findings that excessive Stat3 activity blunted TGF-β-induced signalling (*i.e.* Smad2 phosphorylation) and transcriptional response (*i.e. pCAGA*_*12*_*-luc* activity) in mouse embryo fibroblasts and gastric epithelium of *gp130*^*757F*^ mice due to enhanced transcriptional induction of the TGF-β signalling antagonist Smad7 (Jenkins et al, 2005a).

### Fibrosis in *gp130*^*757F*^ mice occurs independently of Smad3

**Figure 4:**
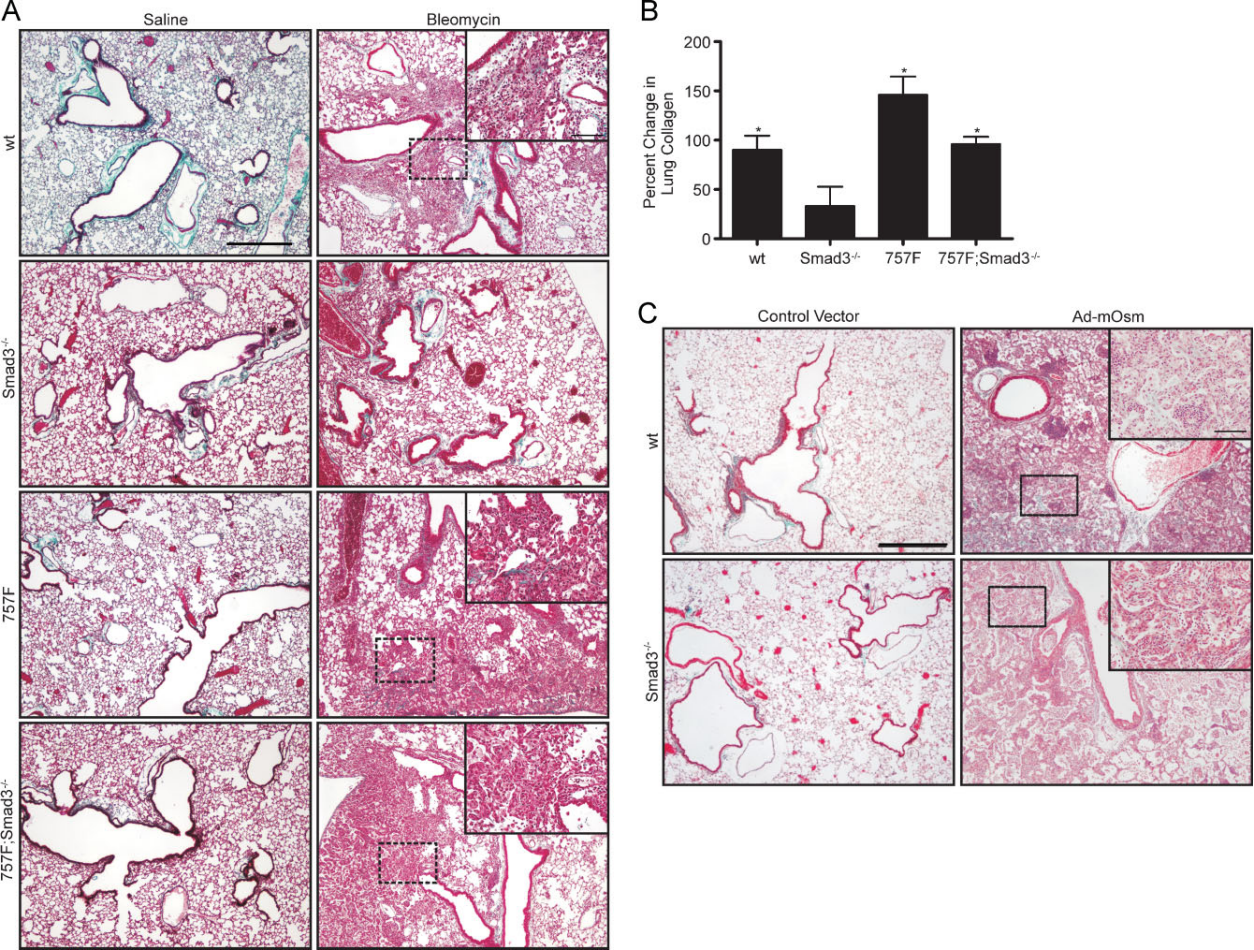
Stat3 promotes lung fibrosis and collagen synthesis independent of Smad3 Masson's trichrome stained sections of lung from *gp130*^*wt*^ (wt), *Smad3*^−/−^ (Smad3^−/−^), *gp130*^*757F*^ (757F) or *gp130*^*757F*^;*Smad3*^−/−^ (757F;Smad3^−/−^) mice 21 days after challenge with bleomycin or saline. Images are representative of *n* = 3 mice. Scale bar = 500 µm (= 100 µm insets).Percent change in collagen content in lung homogenates between saline and bleomycin treated mice of the indicated genotypes 30 days after challenge. *n* ≥ 3 mice. The range of collagen levels between saline and bleomycin treated mice was 4.369–26.48 mg. Data are expressed as mean percentage change ± SEM, with *p < 0.05 using Bonferroni multiple comparisons test.Masson's trichrome stained section of lungs from wt (top) or Smad3^−/−^ (bottom) mice 14 days after trans-nasal delivery of control (left) or Ad-mOsm virus (right). Images are representative of three mice. Scale bar = 500µm (= 100 µm insets).

Since *gp130*^*757F*^ mice develop a more profound fibrotic response to bleomycin despite their attenuated TGF-β responsiveness, we next determined genetically whether their enhanced lung fibrosis could occur independently of canonical TGF-β signalling. We therefore challenged *gp130*^*757F*^ and *gp130*^*757F*^;*Smad3*^−/−^ mice with bleomycin and observed 21 days later in both genotypes of mice a profound fibrotic response that was characterized by excessive hydroxyproline accumulation and collagen deposition in the pulmonary interstitium (Fig 4A and B). In *gp130*^*wt*^ mice, however, ablation of *Smad3* (*Smad3*^−/−^) reduced bleomycin-dependent lung fibrosis in mice as previously reported (Bonniaud et al, 2004; Zhao et al, 2002) when compared to the fibrotic lesions and excessive collagen deposition observed in Smad3 proficient wild-type mice 30 days after bleomycin challenge (Fig 4A and B).

To ascertain that excessive Stat3 activation could mediate bleomycin-induced fibrosis independently of Smad3, we extended this observation to a physiologically more relevant setting where excessive Stat3 signalling occurred in *gp130*^*wt*^ mice in response to prolonged exposure to gp130 cytokines rather than through engagement of mutant *gp130*^*757F*^ receptors. Since prolonged exposure of mice to Osm and other gp130-cytokines promotes excessive collagen production and fibroblast proliferation (Mozaffarian et al, 2008; Scaffidi et al, 2002), we transnasally administered a replication-deficient adenovirus encoding murine *Osm* to wild-type mice and demonstrated an increase in transcriptionally active phosphorylated Stat3 (Supporting Information Fig S6D). Furthermore, continuous exposure of *Smad3*^−/−^ mice to Osm induced severe sub-epithelial and interstitial pulmonary fibrosis 14 days later that was comparable to the response seen in wild-type mice (Fig 4C). This was in contrast to Smad3 deficiency conferring protection from bleomycin-induced lung fibrosis in *gp130*^*wt*^ mice (Fig 4A and B).

### Excessive STAT3 phosphorylation in human IPF tissue

**Figure 5:**
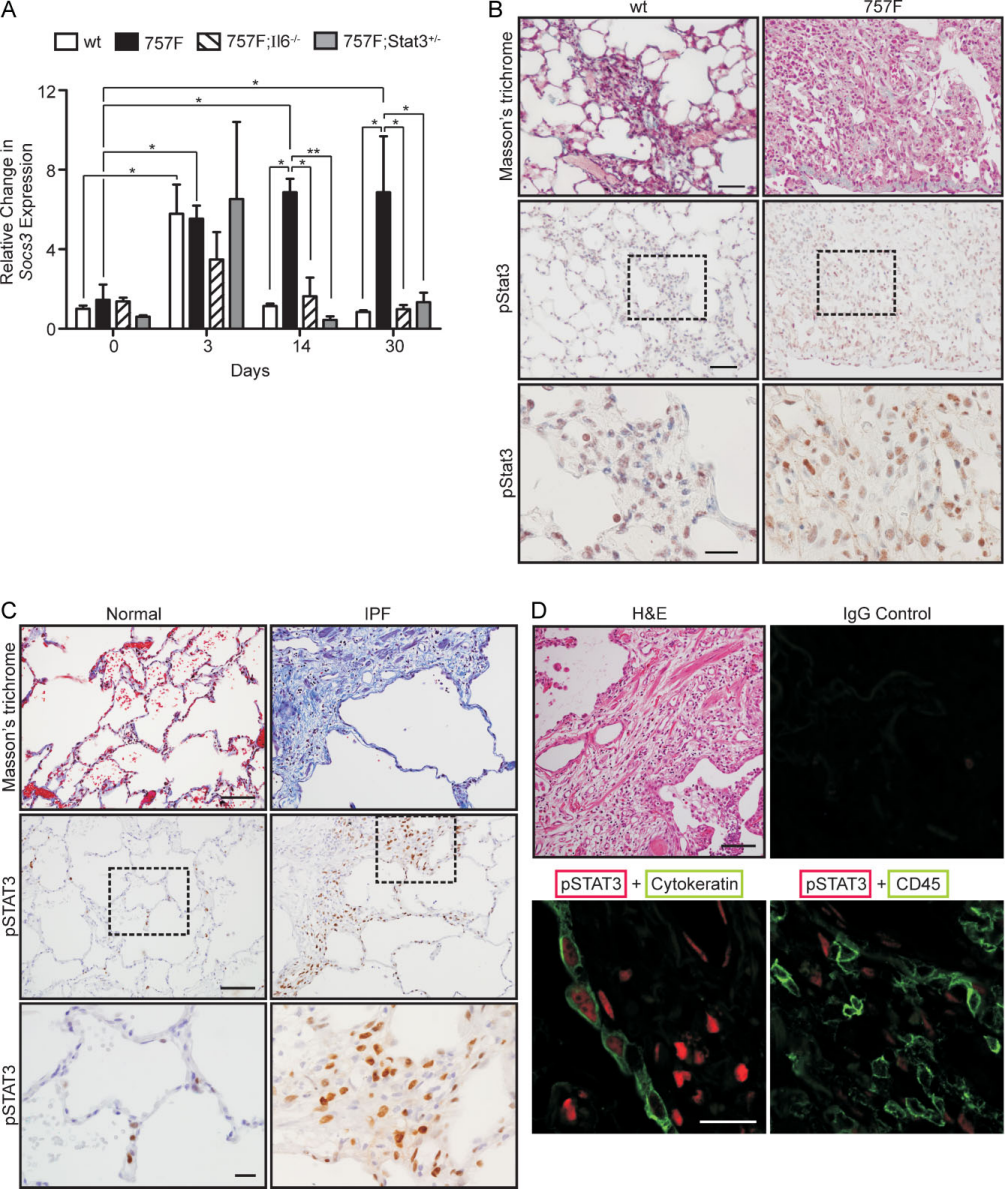
Lung fibrosis is associated with activation of gp130-Stat3 signalling cascade **A.** qPCR analysis of *Socs3* mRNA expression in lung homogenates from *gp130*^*wt*^ (wt), *gp130*^*757F*^ (757F); *gp130*^*757F*^;*Il6*^−/−^ (757F;Il6^−/−^) and *gp130*^*757F*^;*Stat3*^+/−^ (757F;Stat3^+/−^) mice 3, 14 and 30 days after bleomycin challenge or from control mice (0). *Socs3* signals were normalized to *18S* and expressed relative to saline-treated mice of the same genotype. *n* ≥ 3 mice. Data are expressed as mean ± SEM with *p < 0.05 and **p < 0.01 using Bonferroni multiple comparisons test.**B, C.** Adjacent lung sections stained either with Masson's trichrome or phosphorylated Stat3 (pStat3) of lungs from *gp130*^*wt*^ or *gp130*^*757F*^ mice 30 days after bleomycin challenge (**B**), or from IPF patients diagnosed with usual interstitial pneumonitis (IPF-UIP) (**C**). The boxed areas in the middle panels are magnified in the bottom panels showing immunoreactive pStat3 staining associated with fibrotic areas. Images are representative of three mice and four patients, respectively. Scale bar = 100 µm (top and middle panels), = 20 µm (bottom panels).**D.** Haematoxylin and eosin (H&E) stained and dual fluorescence immunohistochemical labelled pSTAT3 (red) and pan-cytokeratin (green) or CD45 (green), respectively, in lung sections from IPF-UIP patients. Scale bar = 100 µm (H&E), = 20 µm (immunofluorescence).

To explore the potential relevance of our findings in the *gp130*^*757F*^ mouse model to human IPF, we first confirmed that excessive Stat3 activation in fibrotic lungs of bleomycin challenged *gp130*^*757F*^ mice was dependent on IL-6 by monitoring expression of the *bona fide* Stat3 target gene *Socs3* (Kidder et al, 2008; Snyder et al, 2008). While we observed an IL-6-independent transient increase in Socs3 expression 3 days after the bleomycin challenge (Fig 5A), *Socs3* expression remained elevated up to 30 days only in IL-6-proficient *gp130*^*757F*^ mice if Stat3 expression was not genetically depleted (*i.e.* in *gp130*^*757F*^;*Stat3*^+/−^ mice). These observations correlated with our findings of accumulation of phosphorylated Stat3 within cells that collectively comprise the fibrotic areas of either bleomycin-challenged *gp130*^*757F*^ or *gp130*^*wt*^ mice, which also extended to cells immediately adjacent to the collagenous deposits (Fig 5B). Next, we retrospectively investigated STAT3 activation in lung sections from IPF patients that were clinically diagnosed with usual interstitial pneumonia (IPF-UIP, Supporting Information Table T1). Since we detected pronounced pSTAT3 staining in the parenchymal cells adjacent to collagenous foci in these biopsies (Fig 5C), we categorized these cells by concordant immunoreactivity for pan-cytokeratin or CD45 as being of epithelial and haematopoietic origin, respectively. This analysis revealed pSTAT3 nuclear staining in cells co-expressing pan-cytokeratin in addition to extensive regions of cells with nuclear pSTAT3 staining that was discordant with cells staining for pan-cytokeratin or CD45 (Fig 5D). These observations are consistent with emerging reports suggesting that STAT3 activation not only occurs within inflammatory cells associated with fibrotic lesions (Lim et al, 2006; Lim et al, 2009; Ogata et al, 2006), but also may play a role in non-epithelial cells to exacerbate the wound-healing responses that are characteristic for IPF lesions.

### Genetic restriction of Stat3 signalling ameliorates the fibrotic response in bleomycin-challenged mice

**Figure 6:**
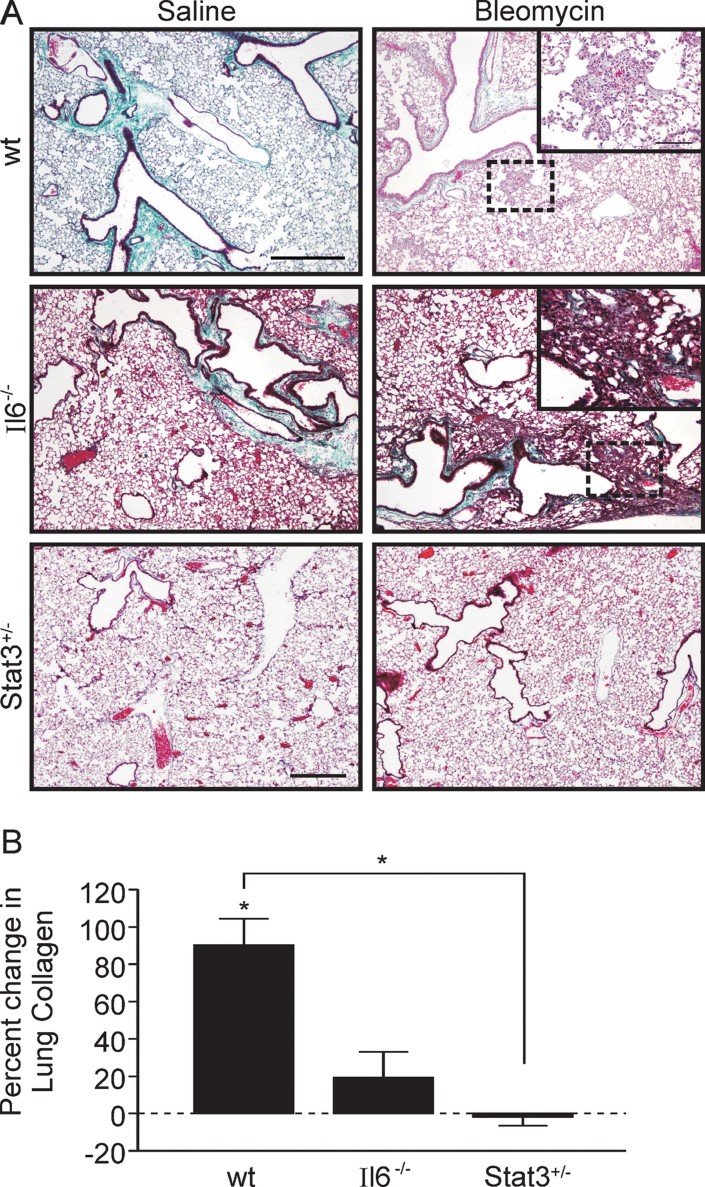
Stat3 haploinsufficiency prevents lung fibrosis Masson's trichrome stained section of lungs for wild-type (wt), *Il6*^−/−^ (Il6^−/−^) and *Stat3*^+/−^ (Stat3^+/−^) mice 30 days after saline or bleomycin treatment. Images are representative of two mice. Scale bar = 500 µm (= 100µm insets).Percent change in hydroxyproline content in lung homogenates between saline and bleomycin-treated mice of the indicated genotypes 30 days after challenge. Data are expressed as mean percentage change ± SEM, with *p < 0.05. *n* = 5 mice. The range of collagen levels between saline and bleomycin treated mice was 4.442–14.070 mg.

The potential role of excessive gp130-mediated STAT3 signalling in human IPF prompted us to explore whether reduction of Stat3 could confer a prophylactic protection from lung fibrosis. To this end, we reasoned that future therapeutic interventions may confer a systemic rather than lung-specific effect on Stat3 activation. We therefore mimicked this situation by testing the susceptibility of *Stat3*^+/−^ mice to bleomycin-induced lung fibrosis. Surprisingly, *Stat3*^+/−^ mice were completely protected from the fibrotic response and associated deposition of collagen in the lung when compared to wild-type mice (Fig 6A and B). Indeed, the prophylactic effect afforded by genetic restriction of Stat3 expression was more effective than complete gene ablation of IL-6 in mice, which only resulted in partial attenuation of the severity of bleomycin-induced fibrosis (Fig 6A and B; Saito et al, 2008). These findings suggest that in addition to IL-6, other gp130 cytokine family members may contribute to the fibrotic response and therefore systemic inhibition of gp130/Stat3 signalling may provide additional benefits that are not afforded through targeting of a single cytokine only.

## DISCUSSION

Although a timely resolution of acute inflammation in response to (bleomycin-induced) injury enables restoration of normal tissue architecture, an unrelenting inflammatory response undermines the healing process and culminates in tissue fibrosis (Wilson & Wynn, 2009; Wynn, 2007). Previous studies have attempted to experimentally replicate excessive inflammation by overexpressing inflammatory cytokines including TNF-α or IL-1β (Kolb et al, 2001; Sime et al, 1998). Here, we provide a model where the increased sensitivity of mutant *gp130*^*757F*^ receptors mediates cytokine-dependent Stat3 activation in mice akin to a smouldering asymptomatic inflammation triggered by prolonged intranasal administration of Osm to wild-type mice or excessive abundance of IL-6 or OSM associated with the pathogenesis of fibrosis in IPF patients (Lesur et al, 1994; Mozaffarian et al, 2008; Xing et al, 1994).

While the exact mechanism of action by which excessive Stat3 activation promotes fibrosis remains to be further elucidated, our observation in bone-marrow reconstituted mice indicates a shared contribution of non-haematopoietic (most likely pulmonary epithelium and fibroblast) and haematopoietic cells types. Furthermore, our data also suggests that excessive Stat3 activation promotes tissue retention of innate immune cells and increases the numbers of immature and mature lymphocytes in *gp130*^*757F*^ mice (Jenkins et al, 2005b). Consistent with this, others have reported that blocking the interaction between the Stat3 target gene *Icam-1* and lymphocytes decreased lymphocyte retention in the normal pulmonary vasculature (Klemm et al, 2000). Although *IL6* is a well characterized inflammatory target gene for TNF-α, and bleomycin-induced fibrosis is ameliorated in *Tnfr*^−/−^ mice (Ortiz et al, 1998), B cell infiltrates also fuel IL-6-mediated and Stat3-dependent cancer growth following the release of lymphotoxin (LT) (Ammirante et al, 2010). Intriguingly, bleomycin-induced fibrosis is not only prevented in *Tnf*^−/−^;*Ltα*^−/−^ mice (Piguet et al, 1997), but also depends on the presence of CD19-positive B-cells in wild-type mice (Komura et al, 2008). Consistent with these observations, we provide here genetic evidence that the bleomycin-induced fibrotic response in *gp130*^*757F*^ mice requires mature B lymphocytes. This correlates with exacerbated accumulation of B220+ cells and with enhanced abundance of the Th2 cytokine IL-13 in the lung, which in itself not only requires Stat3 for its effective production (Stritesky et al, 2011), but also promotes fibrosis (Fichtner-Feigl et al, 2006). It is therefore tempting to speculate that, for instance, the combined activity of LT and TNF-α, through induction of gp130-activating cytokines, indirectly promotes Stat3 signalling and the excessive matrix production that underpins lung fibrosis. This view is consistent with observations that the lower respiratory tract of IPF patients shows excessive TNF-α, LT-α and IL-6 expression (Lesur et al, 1994; Pantelidis et al, 2001) and that the fibrotic response correlates with B lymphocyte accrual in lungs from non-specific interstitial pneumonia patients (Keogh & Limper, 2005). Furthermore, exogenous administration of IL-6 family cytokines may short-circuit the need for lymphocytes, because in their absence in *Rag1*^−/−^ mice, Osm can promote lung fibrosis (Mozaffarian et al, 2008). Indeed, IL6-dependent Stat3 activation also promotes the production of two other potential therapeutic targets for bleomycin-induced fibrosis, namely miR-21 and the generation of IL-17A-producing cells (Liu et al, 2010; Wilson et al, 2009). Together with our observations of excessive pSTAT3 accumulation in human IPF biopsies, these recent findings further strengthen a rationale to exploit Stat3 as an attractive signalling node for novel therapeutic strategies for IPF.

Here, we provide genetic evidence for the capacity of Stat3 to link signalling from IL-6 family cytokines to stimulation of *Col1a1* expression and parenchymal collagen deposition independently of canonical TGF-β/Smad3 signalling. Previous reports have described the capability of IL-6 to stimulate collagen deposition in the skin and have identified putative Stat3 binding sites in the *Col1a1* and *Col3a1* promoters (Lim et al, 2006). Consistent with this, we observed reduced bleomycin-induced fibrosis in the absence of IL-6 and that gp130-mediated increase in collagen gene reporter activity in fibroblasts was dependent on Stat3. By contrast, transgenic expression of IL-6 in the Clara cells of the uninjured mouse lung was associated with airspace enlargement in older mice (Kuhn et al, 2000), reminiscent of the distinct emphysematous and inflammatory changes in lungs of naïve 6 months old *gp130*^*757F*^ mice (Ruwanpura et al, 2011). Although the *gp130*^*757F*^ mice used in this study were less than 3 months old and occasionally showed mild air space enlargement (Fig 1; Ruwanpura et al, 2011), there was no evidence for increased IL-6 levels (Fig 2D and Supporting Information Fig S4A) at this stage. Since IL-6 expression rapidly increased after bleomycin challenge, we surmise that excessive IL-6 within the injured lung promotes fibrosis.

Although the development of experimental lung fibrosis in wild-type mice is inhibited in the absence of canonical TGF-β/Smad3 signalling (Bonniaud et al, 2004; Zhao et al, 2002), the protective effect arising from its ablation is overcome in situations of excessive Stat3 activation that results from mutant *gp130*^*757F*^ receptors during bleomycin-induced lung injury or from the sustained presence of IL-6 family cytokines, including Osm. Although the inflammatory response elicited by systemic LPS administration induces the production of the transcriptionally active Smad2 splice variant Smad2^ΔExon3^ (Dunn et al, 2005), which can replicate Smad3 transcriptional activity (Yagi et al, 1999), it remains unknown whether Smad2^ΔExon3^ can mediate activation of the *Col1a1* gene promoter. Notwithstanding that canonical TGF-β signalling integrates various inflammatory and regenerative stimuli that promote wound healing and the deposition of *de novo* tissue matrix, our data provide a strong rationale for targeting the gp130/Stat3 signalling axis as a complementary approach to current clinical trials focusing on components of the TGF-β signalling cascade.

## MATERIALS AND METHODS

### Mice and treatments

All mutant *gp130*^*ΔStat*^ and *gp130*^*757F*^ mice along with their corresponding *Stat3*^+/−^, *Stat1*^−/−^, *Il6*^−/−^, *Rag1*^−/−^, *µMT*^−/−^ or *Smad3*^−/−^ counterparts were between the ages of 8 and 12 weeks and were propagated on a mixed 129/Sv × C57BL/6 genetic background (Ehlich et al, 1993; Ernst et al, 2008; Jenkins et al, 2005a; Tebbutt et al, 2002). All animals were housed under specific pathogen-free conditions and experimentation was approved by the Institute's Animal Ethics Committee.

We transnasally delivered a bolus of 50 µl bleomycin (0.05 U/mouse; Blenoxane, Bristol-Myers Squib, New York, USA), or 2 µg HYPER-IL-6 every second day for 14 days to anaesthetized mice. We trans-nasally delivered 5 × 10^7^ PFU adenoviral vector in 30 µl PBS and collected lungs 14 days later (Langdon et al, 2003). Lethally irradiated mice were reconstituted with bone marrow (Ernst et al, 2008) at least 30 days before challenging with bleomycin. BAL fluid was collected by endotracheal instillation of three 0.4 ml aliquots of PBS to recover approximately 1 ml of fluid, pelleted cells were stained with Quik-Dip (Scot Scientific, Taren Point, Australia) and differential cell counts performed.

**The paper explained**

PROBLEM:

Idiopathic pulmonary fibrosis (IPF) is a heterogeneous disease with an incidence of approximately 1 per 10,000, equivalent to many cancers, and which is unresponsive to therapy and fatal in outcome. Although fibrosis is thought to arise from excessive tissue response to injury, the molecular mechanisms underlying the initiation of IPF remain largely unknown.

RESULTS:

In the present study we utilize mutant mice carrying engineered mutations in gp130, the interleukin (IL)-6 co-receptor, to provide genetic evidence for the causal involvement of IL-6-dependent Stat3 signalling in this disease in an established preclinical setting. Using a genetic complementation approach, we define a functional requirement for IL-6, Stat3 and mature B-lymphocytes for the development of disease. Significantly, we provide genetic and biochemical evidence that Stat3-driven lung fibrosis can occur by a mechanism independent of signalling through the canonical transforming growth factor (TGF)-β/Smad3 pathway. We also document excessive Stat3 activation as a common feature in human patients with IPF and provide evidence that reduction of systemic Stat3 expression in mice decreases susceptibility to bleomycin-induced fibrosis.

IMPACT:

Previous studies have demonstrated that canonical TGF-β/Smad3 signalling is pivotal to the pathogenesis of pulmonary fibrosis. Our study demonstrates that that therapeutic targeting of IL-6/Stat3 signalling and/or B-lymphocytes may ultimately afford more efficacious treatments for IPF and related diseases than those directed solely against TGF-β/Smad3 signalling.

### Cytokines, plasmids and antibodies

The *pCol1a1-luc* and *p*(*CAGA*)_*12*_*-luc* constructs and the production of HYPER-IL-6 has been described (Buttner et al, 2004; Fischer et al, 1997). Recombinant human IL-6 and human TGF-β1 were from Bender Medsystems (Vienna, Austria) and Sigma–Aldrich (St Louis, USA), respectively. Antibodies directed against B220 were from Becton Dickinson Biosciences (San Jose, USA), CD3 from Serotec (Kidlington, UK), F4/80 from Abcam (Cambridge, UK), α-tubulin (Sigma–Aldrich), CD45 and cytokeratin AE1/AE3 from Dako (Glostrup, DK), phosphorylated Stat3 (Cell Signaling Technology, Danvers, USA) and β-actin, Stat3 or phosphorylated Smad3 (Santa Cruz Biotechnology).

### Collagen quantification, lung fibroblast preparation and cellular assays

We analysed hydroxyproline content of lung homogenates as described (Mutsaers et al, 1998). We digested lungs in 0.25% trypsin-EDTA (Invitrogen Life Technologies, Carlsbad, USA) at 37°C for 30 min and removed remaining connective tissue. Digests were finely minced and incubated in 1 mg/ml collagenase 1 (Invitrogen Life Technologies) for 1 h at 37°C. Pelleted cells were used for establishing primary lung fibroblast cultures in DMEM (Invitrogen Life Technologies) supplemented with 10% foetal bovine serum (Sigma–Aldrich, Castle Hill, Australia), 4 mM l-glutamine (Invitrogen Life Technologies) and penicillin–streptomycin–fungizone cocktail (Invitrogen Life Technologies), and assays were performed on passage 3–5 cells. We exposed embryonic fibroblasts, co-transfected with *pCol1a1-luc* and the *pCMV-Rluc* plasmid to HYPER-IL-6 and determined dual luciferase activity in triplicate cultures 48 h later (Jenkins et al, 2005a).

### RNA isolation and expression analysis

We extracted total RNA from tissues or snap-frozen cell cultures with TRIzol (Invitrogen Life Technologies) and prepared cDNA from 1 µg of total RNA using the SuperScript III System (Invitrogen Life Technologies). We performed quantitative RT-PCR analysis on lung tissues in triplicate with the iCycler platform (Bio-Rad Laboratories, USA) using SYBR Green (Invitrogen), and quantified *Col1a1* (forward 5′-GGAAGAGCGGAGAGTACTGG-3′, reverse 5′-GTACTCG AACGGGAATCCAT-3′), *Il6* (forward 5′-GTATGAACAACGATGATGCACTTG-3′, reverse 5′-ATGGTACTCCAGAAGACCAGAGGA-3′), *Il11* (forward 5′-CTGCACAGATGAGAGACA AATTCC-3′, reverse 5′-GAAGCTGCAAAGATCCCAATG-3′) *Osm* (forward 5′-AACACTGC TCAGTTTGACCCTCAGT-3′, reverse 5′-AGGTTTTGGAGGCGGATATAGGGCT-3′), *Socs3* (forward 5′-GCGGGCACCTTTCTTATCC-3′, reverse 5′-TCCCCGACTGGGTCTTGAC-3′) transcripts using glyceraldeyhde-3-phosphate-dehydrogenase (*Gapdh*) (forward 5′-TCGG TGTGAACGGATTTGGC-3′, reverse 5′-GAATTTGCCGTGAGTGGAGT-3′) or *18S* (forward 5′-GTAACCCGTTGAACCCCATT-3′, reverse 5′-CCATCCAATCGGTAGTAGCG-3′) as a housekeeping gene (Scaffidi et al, 2002). Each RNA sample was analysed in duplicate.

### Immunohistochemistry

We stained consecutive 5 µm sections of inflated (250 mm-H_2_O pressure), paraformaldehyde-fixed and paraffin-embedded lungs with haematoxylin and eosin, Masson's trichrome (staining collagenous deposits green), or for the cell lineage markers B220, CD3, F4/80 or phosphorylated Stat3. We characterized inflammatory foci as a collection of granulocytic and/or lymphocytic cells that occupied at least one field of view (20× objective lens), and within these foci counted the total number of indicated cells from three or more randomly chosen sections from each tissue block. Paraffin embedded human tissue was immunolabelled for the cell lineage markers pan-cytokeratin, CD45 and phosphorylated STAT3 following antigen retrieval with 10 mM citrate buffer pH6.

### Patient samples

Tissue biopsies from four male patients with diagnosed IPF-UIP (age range of 59–69 years, mean of 63 years) and appropriate controls were used in this study. Human ethics approval for this study was provided through Bellberry Limited for work carried out at Sir Charles Gairdner Hospital, W.A., Australia. All human samples used in this study were retrospective paraffin embedded tissue samples taken for diagnostic purposes. The collection and use of these samples for this study was consistent with Section 3.2.4 of the National Health and Medical Research Council of Australia, Australian Health Ethics Committee guidelines for research involving humans and Section 25 of the World Medical Association, Declaration of Helsinki.

### Statistical analysis

Data are expressed as mean ± SEM, with *p < 0.05, **p < 0.01, ***p < 0.001. Statistical analysis was performed using one-way analysis of variance and Bonferroni's multiple comparisons post-test.

For more detailed Materials and Methods see the Supporting Information.

## Supplementary material

Detailed facts of importance to specialist readers are published as ”Supporting Information”. Such documents are peer-reviewed, but not copy-edited or typeset. They are made available as submitted by the authors.
