# Supplementary material for: Genetic partitioning of interleukin-6 signalling in mice dissociates Stat3 from Smad3-mediated lung fibrosis
Source: EMBO Mol Med. 2012 Jun 8;4(9):939–51. doi: 10.1002/emmm.201100604 (PMC3491826; doi:10.1002/emmm.201100604)
Supplement: Supplementary file 1 [file emmm0004-0939-SD1.pdf]

EMBO
Molecular Medicine

Manuscript EMM-2011-00604

Genetic partitioning of interleukin-6 signalling in mice dissociates Stat3 from Smad3-mediated lung fibrosis

Robert J. J. O'Donoghue, Darryl A. Knight, Carl D. Richards, Cecilia M. Prêle, Hui Ling Lau, Andrew G. Jarnicki, Jessica Jones, Steven Bozinovski, Ross Vlahos, Stefan Thiem, Brent S. McKenzie, Bo Wang, Philip Stumbles, Geoffrey J. Laurent, Robin J. McAnulty, Stefan Rose-John, Hong Jian Zhu, Gary P. Anderson, Matthias R. Ernst, Steven E. Mutsaers

Corresponding author: Matthias Ernst, Ludwig Institute

Review timeline:

Submission date:	17 June 2011
Editorial Decision:	10 August 2011
Revision received:	24 November 2011
Editorial Decision:	17 January 2012
Rebuttal:	17 January 2012
Editorial Correspondence:	02 February 2012
Editorial Decision:	12 March 2012
Revision received:	16 April 2012
Accepted:	09 May 2012

Transaction Report:

(Note: With the exception of the correction of typographical or spelling errors that could be a source of ambiguity, letters and reports are not edited. The original formatting of letters and referee reports may not be reflected in this compilation.)

1st Editorial Decision 10 August 2011

Thank you for the submission of your manuscript "Genetic partitioning of interleukin-6 signalling in mice dissociates Stat3 from Smad3 mediated lung fibrosis" to EMBO Molecular Medicine and please accept my apologies for the delayed reply We have now finally heard back from the three referees whom we asked to evaluate your manuscript. You will see that they find the topic of your manuscript potentially interesting. However, they also raise significant concerns on the study, which should be addressed in a major revision of the manuscript.

Importantly, both Reviewers #2 and #3 point out that the same controls seemed to have been used in Figures 2 and 5 and that it is crucial that additional controls are performed and the quality of the histology is improved. Reviewer #1 highlights that, in addition to addressing apparent inconsistencies with previously published data, the potentially confounding issue of spontaneous lung inflammation and emphysema in 757F mice needs to be addressed. Of note, Reviewer #2 points out that the dynamic development of fibrosis should be assessed.

Given the balance of these evaluations, we feel that we can consider a revision of your manuscript if you can convincingly address the issues that have been raised within the time constraints outlined below.

Revised manuscripts should be submitted within three months of a request for revision. They will otherwise be treated as new submissions, unless arranged otherwise with the editor.

I look forward to seeing a revised form of your manuscript as soon as possible.

Yours sincerely,

Editor
EMBO Molecular Medicine

***** Reviewer's comments *****

Referee #1:

The manuscript by O'Donoghue et al reports the use of a series of genetically modified mice with altered levels of Stat signaling by IL6 family cytokines to further uncover fibrotic mechanisms in IPF. They use a well documented bleomycin induced lung fibrosis model, and overall the manuscript is well written and data clearly presented. The concept that IL6 signals promote lung fibrosis is not new, but instead the novelty of this study lies with attempts to dissociate the gp130-Stat3 pathway from that of TGFb. While the manuscript was an interesting read, there are several inconsistencies with the data as presented that need to be addressed prior to acceptance. Also some aspects of this study are similar to previously published work on the role of IL6 and Stats (eg Stat1) in bleomycin-fibrosis and yet data presented are not consistent with such studies, and these discrepancies needs to be expanded upon. In addition some of the conclusions made were speculative and not appropriately supported by the data presented, and require additional data to be generated.

Specific comments:

It appears the gp130757F and g130dSTAT mice have been widely documented already in the literature. Accordingly I found Fig 1 somewhat unnecessary and more suited to a supplementary Figure.

Fig 2: the lungs of gp130757F mice and g130dSTAT mice with saline looks different to each other and wild-type lungs. The 757F mice appear the same as those recently reported (Ruwanpura et al. Am J Respir Cell Mol Biol. 2011 Feb 17. [Epub ahead of print]) to develop in a spontaneous manner lung inflammation and emphysema. The authors need to address this issue which potentially confounds their conclusions. It is unclear what were the age of mice used in this study? Also it would appear that at least the 757F mice with saline shown in Fig 2 may have this lung abnormalities already (eg airspace enlargement).

Can the authors provide further detail of what they mean by the statement on page 8 that "bleomycin challenged gp130757F mice showed extensive changes to their lung architecture".

It has previously been shown that bleomycin-induced fibrosis is more severe in Stat1-/- mice (Walters et al. Am J Pathol. 2005;167(5):1221-9) so it is not surprising that "Stat1 ablation in gp130757F;Stat1-/- mice failed to reduce the excessive pulmonary fibrosis". One of the mechanisms proposed for this severe fibrosis was increased Stat3 activation seen in Stat1-/- mice, although Stat3 induced by IL6 cytokines was not examined. Therefore one would predict fibrosis may be worse in the gp130757F;Stat1-/- mutant mice: was this the case? What are the levels of collagen in the lungs of treated gp130757F;Stat1-/- mice? It is important to address this point, and assess the activation of Stat3 in gp130757F;Stat1-/- mice because if elevated, then consistent with the hypothesis of O'Donoghue et al that Stat3 drives fibrosis, fibrosis should be worse in gp130757F;Stat1-/- mice.

The authors state that Supp Fig S2a shows "genetic ablation of Il6 in gp130757F;Il6-/- mice ameliorated the excessive fibrotic response induced in bleomycin challenged gp130757F mice". From the sections shown it is difficult to see if they are referring to a complete or partial protection from bleomycin-induced fibrosis, and how this relates to level of protection previously observed in bleomycin-treated Il6-/- mice (Saito et al Am J Respir Cell Mol Biol. 2008;38(5):566-71). They should therefore also show the level of collagen in the lungs of gp130757F;Il6-/- mice as they have done in Fig 3a and b for other mice.

In Supp Fig 2 the authors look at the production of several cytokines of untreated and treated mice, but they don't look at the production of other IL6 family cytokines. This is an oversight since it is stated in the introduction that "transgenic overexpression of IL-11 or Oncostatin M (Osm) in mice, for instance, promotes lung scarring with striking histopathological similarities to that observed in human disease" (ie pulmonary fibrosis). Also Osm is used in Fig 5 to induce fibrosis in WT mice, and the last paragraph of the results section also suggests other IL6 related cytokines may be playing a role. The authors must therefore measure the production of these and related IL-6 cytokines which could also play a role in disease in this mouse model.

What are the Stat3 and Stat1 activation level like in the lungs of bleomycin-treated gp130757F;Il6-/- mice? Since the authors propose that Stat3 promotes fibrosis, then the level of Stat3 in these treated mice should be lowered.

Since Il6-/- mice has been used to show that IL6 is important for neutrophil (and macrophage) infiltration in response to bleomycin (Saito et al Am J Respir Cell Mol Biol. 2008;38(5):566-71), data from gp130757F;Il6-/- mice (protected from fibrosis) should also be included in Fig 4a and b as a comparison. In fact inflammatory cell numbers were reduced in both BALF and lung tissue in treated Il6-/- mice (protected from fibrosis) compared to WT mice as shown by Saito et al, which potentially contradicts the findings in Fig 4 that gp130757F;Stat3+/- (reduced fibrosis to wild-type levels) and gp130dStat mice (protected from fibrosis) have increased BAL fluid and lung tissue inflammatory cells numbers compared to WT. It is also unclear that if as the authors say "bleomycin-induced lung fibrosis depends on the preceding inflammatory response (Moeller et al., 2008)", then why is the lymphocytes accumulation in the lung parenchyma (which is supposed to correlate with fibrosis severity) in Fig 4b and Supp Fig S3 similar between gp130757F;Stat3+/- and gp130dStat mice despite these mice displaying differing levels of fibrosis (as shown in Fig 2).

Move Fig 4d to Supp Fig S3.

In Supp Fig S4, if as authors say Stat3 activation is reduced to wild type levels in gp130757F;Stat3+/- mice, and Stat3 influences TGFb signaling, then why is there a noticeable stronger level of pSmad3 in gp130757F;Stat3+/- compared to WT mice? It would be preferable to include gp130dStat mice in this Fig as presumably they don't show any activation of Stat3 from gp130 and this may yield a more concise result as opposed to partial suppression of gp130-Stat3 signal in gp130757F;Stat3+/- mice.

In Fig 5, I found the use of Osm, while compelling in inducing fibrosis, somewhat confusing since earlier in the manuscript (and again in Fig 5e) the authors have focussed on IL6 as a key cytokine from this family driving Stat3 activation and disease in this mouse model. Is Osm playing a role in the gp130757F lung fibrosis phenotype? Also Osm is used to induce excessive Stat3 signaling in WT mice, yet there is no evidence provided that Stat3 activation in the lung is substantially increased in WT mice with the Osm exposure. The authors must address this.

The statement relating to Smad2 Exon3 expression that" we observed an increase in its expression between bleomycin-challenged wild-type and gp130757F mice (Figure 5d) is misleading because Smad2 Exon3 expression is reduced in bleomycin-challenged WT mice compared to all other experimental groups. Remove or reword sentence. Similarly this is referred to in the Discussion, and it is unclear what data supports the latter part of the sentence "we observed increased abundance of Smad2 Exon3-specific mRNA in bleomycin-challenged gp130757F compared to gp130wt mice, which was further exacerbated in gp130757F mice in an IL6/Stat3-dependent manner". Very importantly too, if a functional effect of altered Smad2 Exon3 mRNA levels is to be assigned to the increased fibrotic response of gp130757F mice and this relates to Stat3, then the authors must include expression data in Fig 5d for Smad2 Exon3 in the other gp130dStat, gp130757F;Stat3+/- and gp130757F;Il6-/- mice. If this correlation is true, then one would predict Smad2 Exon3 expression to be down in protected mice.

The data for Smad2dExon3 splice variant is not convincing, and the statement that "Since the formation of transcriptionally active pSmad3 is impaired in gp130757F lung fibroblasts (Supplementary Figure S4b), this result is likely to reflect the effect of increased production of the Smad2 Exon3 protein" is purely speculation because no protein expression data for the splice variant

is shown. I assume antibodies against Smad2 can be used to detect this variant which would run at a different size to full-length Smad2?

Referee #2 (Comments on Novelty/Model System):

This submission is derived from a well-established group of investigators using appropriate model systems for the question at hand, albeit the authors rely on one model in particular, the bleomycin model. The observation and interdependence of IL-6, STAT3, and TGF-beta signaling is novel in lung fibrosis, yet not entirley novel in fibrotic diseases. While the authors provide some data on human IPF samples (Fig. 6b), these data are very limited, thus questioning the medical impact.

Referee #2 (Other Remarks):

This reviewer has not been part of the initial decision/revision, thus may not be completely aware of previous questions/concerns.

In general, this is a well-written and, for the most part, well-prepared report on the interdependence of STAT3, IL-6, and TGF-beta signaling in experimental lung fibrosis. The report heavily relies on complex transgenic mice, and the comparison of these during bleomycin-induced lung fibrosis. Given the caveat that these complex genetic alterations will most certainly (and have been described to do so) exhibit alterations to more than one signaling pathway, it is in general very hard to conclusively deduce specific defects in a specific molecule using these mice.

Specifically, I have the following suggestions for improvement:
1. The different mice are all compared at one time-point (30 days post bleomycin application). This limits the analysis in that the dynamic development of fibrosis is not assessed and depicted. Is the difference a real difference of fibrosis, or rather earlier/later onset, or earlier/later resolution. This needs clarification.
2. The authors report using Bleomycin at 0.05U/kg BW. This is two potencies lower than most other users.
3. The authors argue that fibrosis in 757F mice occurs independently of Smad3, yet they present evidence that an alternatively spliced Smad2 may be involved. This is a key issue and needs to be addressed. Protein analysis and phosphorylation of Smads should be addressed, as mRNA expression levels do not correctly assess activation.
4. Finally the authors have duplicated Figures in the submission (Fig. 2, top lane is identical to Fig. 5a, top lane). This raises the question whether one positive control of bleomycin has been used for all experiments. If yes, this is a clear limitation and addtl. controls need to be performed.

Referee #3 (Comments on Novelty/Model System):

Bleomycin-induced lung fibrosis is a well accepted model for IPF in humans

Referee #3 (Other Remarks):

In "Genetic partitioning of interleukin-6 signalling in mice dissociates Stat3 from Smad3 mediated lung fibrosis," O'Donoghue et al. provide genetic, in vivo evidence that gp130/Stat3 signaling is important for the formation of bleomycin-induced pulmonary fibrosis in mice.
The manuscript is made extremely difficult to read by grammatical errors and leaps of logic that are not always supported by the data presented. While the manuscript includes interesting kernels of data, serious concerns prevent this reviewer from recommending publication without extensive revision.
Some experimental methods are not adequately described. For example, the legend for Figure 4c is not understandable (i.e. timing and logistics of bone marrow transplants). There is a reference to embryonic fibroblasts in the methods section, but no corresponding data in the manuscript.
There are incorrect references given throughout. For example, Cool 2006 and Moodley 2003 do not show that epithelial injury causes the proliferation of fibroblasts and sub-epithelial myofibroblasts

that are associated with excess collagen production.
In some respects, the Stat3 loss-of-function experiments are the most informative. Have the authors performed bone marrow transplants using Stat3 heterozygous mice as donors and hosts? Similarly, the authors could address whether TGFB is sufficient to induce fibrosis in Stat3 heterozygous mice.
How do the authors reconcile their data with the previously published finding that transgenic overexpression of IL6 did not cause pulmonary fibrosis (Kuhn et al AJRCMB 22: 289-295 (2000)? Why is Osm sufficient to cause fibrosis but not IL-6?
Based on previous publications from this group (e.g. Moodle et al 2003), one would expect that constitutive Mapk signaling in fibroblasts would exacerbate pulmonary fibrosis. The opposite finding is reported here (i.e. deltaStat mice were partially protected from bleomycin-induced pulmonary fibrosis). Does administration of IL6 to deltaStat mice cause fibrosis?
The authors conclude from their bone marrow transplant experiments that gp130 signaling in the parenchyma is pro-fibrotic. What are the data to support this conclusion? The data shown in Figure 4 suggest that the pro-fibrotic effects of gp130 might be mediated entirely by myeloid lineages (i.e. 757F hosts reconstituted with deltaStat bone marrow showed a wild type response to bleomycin). Moreover, in naÔve wt mice (Fig4C) and wt mice (Fig4D) why aren't the levels of Collagen increased 21d after bleomycin? This makes it impossible to interpret any data presented in the figure.
Is the histology from bleomycin treated 757F;Rag2null mice normal?
The quality of the histology and micrographs is poor. The trichrome staining is variable and this makes comparison of data difficult (compare 757F saline in Figure 2 and Figure 5). Some images are used as controls in multiple experiments (Figure 2 and 5 wild type, saline and bleomycin). And some important controls are omitted. If pStat3 is increased in IPF, then what does pStat3 staining look like in a normal lung? No conclusions about the cell types in which pStat3 is localized can be drawn from the images in Fig 6. This would require at least double immunofluorescence and preferably confocal microscopic analysis.

1st Revision - Authors' Response 24 November 2011

Issues raised by the Editor

(1) The repeated depiction of wild-type controls in Figures 2a, 6a and 5 and the quality of histology.

We have now repeated experiments with all cohorts of mice (including appropriate wild-type controls) and have generated new micrographs for most of the Masson's trichrome stains to improve the quality of micrographs and standardise staining throughout the manuscript (Fig 1, Fig4a and Fig 6a as well as Supplementary Figures 2b, 3a and 4d).

(2) *The age-related emphysematous changes in lungs of gp130(757F) mice that we previously described in our collaborative study (Ruwanpura et al.; Am. J. Respir. Cell Mol. Biol., 2011).*

We have clarified in the revised Results, Discussion and Methods sections (p7, 16, 18) that the use of younger (2-3 mo old) mice in this study largely alleviates the risk of dealing with potential confounding effect that could arise from the emphysematous changes observed is older (~6mo) mice that we previously reported.

(3) *Documenting the dynamic changes of fibrosis development.*

In Figure 2d we now provide additional data documenting the dynamic development of fibrosis in the gp130(757F) mice.

Reviewer #1

"...I found Fig 1 somewhat unnecessary and more suited to a supplementary figure."

The previous Fig 1 has been moved to the Supplementary data as Supplementary Fig 1.

Fig 2: The 757F mice appear the same as those recently reported (Ruwanpura et al.) ... and to develop in a spontaneous manner lung inflammation and emphysema. The authors need to address this…It is unclear what were the age of mice used in this study? …it would appear that at least the 757F mice with saline shown in Fig 2 may have this lung abnormalities already (eg airspace enlargement).

In the Ruwanpura *et al.* paper we have reported that gp130(757F) mice spontaneously develop mild emphysematous changes at 6 months of age. However, at this age we could not detect inflammatory changes *per se* as neither BAL fluid cellularity nor histological assessment revealed increases in numbers of inflammatory cells, despite increased expression of IL-6 in the lungs of these mice. To avoid a confounding influence of this late-onset phenotype on our assessment of bleomycin-induced lung injury, we used exclusively gp130(757F) mice between 8 and 12 weeks of age for the present study. In the Ruwanpura *et al.* paper we characterize the emphysematous changes in gp130(757F) as (i) increased lung volume; (ii) reduction in the mean linear intercept value; and (iii) the loss of alveoli. Since the photomicrograph in question in our original Figure 2a is only suggestive of a reduction in the mean linear intercept value, we do not believe this to constitute the emphysematous changes we documented in the Ruwanpura *et al.* paper. Accordingly we now show a photomicrograph of an adjacent filed of the same lung as new Figure 1a. We now discuss our present findings in light of our previous findings in the Ruwanpura *et al.* paper in the Discussion section on p17.

…provide further detail of … the statement … that "bleomycin challenged gp130757F mice showed extensive changes to their lung architecture".

This statement was referring to the widespread consolidation of airspaces, thickened alveolar septae, inflammation and epithelial dysplasia of the 757F lungs after bleomycin challenge in general terms. We now explicitly refer to these changes in the Result section on p7.

It has previously been shown that bleomycin-induced fibrosis is more severe in the Stat1-/- mice (Walters et al. Am J Pathol.)*…Therefore one would predict fibrosis may be worse in the gp130757F;Stat1-/- mutant mice: was this the case? What are the levels of collagen in the lungs of treated gp130757F;Stat1-/- mice?*

We now document as additional data increased collagen transcription in bleomycin-challenged gp130(757F);Stat1-/- compound mutant mice compared to bleomycin-challenged wild-type mice (Supplementary Figure S2c), but this remains below the level found in gp130(757F) mice. Therefore our finding does not contradict that by Walters *et al.*, but suggest that the fibrotic response in gp130(757F) mice mediated by excessive Stat3 activation may exceed that mediated by the absence of Stat1 expression. These observations have been clarified in the Result section on p8.

The authors state that Supp Fig 2a shows "genetic ablation of Il6 in gp130757F;Il6-/- mice ameliorated the excessive fibrotic response induced in bleomycin challenged gp130757F mice"…it is difficult to see if they are referring to a complete or partial protection from bleomycin-induced fibrosis, and how this relates to level of protection previously observed in bleomycin-treated Il6-/- mice (Saito et al Am J Respir Cell Mol Biol. 2008; 38(5):566-71). They should therefore show the level of collagen in the lungs of gp130757F;Il6-/- mice as they have done in Fig 3a and b for other mice.

We have included additional data demonstrating reduced levels of collagen gene transcription in the lungs of gp130(757F);Il6-/- mice (Supplementary Figure 3b).

In Supp Fig 2 the authors look at the production of several cytokines of untreated and treated mice, but they don't look at the production of other IL6 family cytokines…The authors must therefore measure the production of these and related IL-6 cytokines which could also play a role in disease in this mouse model.

We have included additional data documented transcriptional levels of the IL-6 cytokines family members IL-11 and Osm (Supplementary Figure 2d), and protein levels for IL6 and LIF (Supplementary Figures 3d and 3e) after bleomycin challenge.

What are the Stat3 and Stat1 activation level like in the lungs of bleomycin-treated gp130757F;IL6-/- mice? Since the authors propose that Stat3 promotes fibrosis, then the level of Stat3 in these treated mice should be lowered.

Unfortunately we were not able to generate sufficient amounts of tissue from lungs of gp130(757F);Il6-/- mice to perform this analysis. However, given our findings that fibrosis in gp130(757F);Il6-/- mice is attenuated to a similar extent as in 757F;Stat3+/- mice and the overwhelming evidence in the literature for the capacity of IL6 to activate Stat3 (and Stat1), we believe it is reasonable to infer that Stat3 activation is likely to be reduced gp130(757F);Il6-/- mice.

...data from 757F;Il6-/- (protected from fibrosis) should also be included in Fig 4a and b as a comparison.

We now include additional data documenting changes to cellularity and composition of BALF following bleomycin challenge of gp130(757F);Il6-/- mice (Supplementary Figures 3c)

... inflammatory cell numbers were reduced in both BALF and lung tissue in treated Il6-/- mice (protected from fibrosis) compared to WT mice as shown by Saito et al, which potentially contradicts the findings in Fig 4 that gp130757F;Stat3+/- (reduced fibrosis to wild-type levels) and gp130dStat mice (protected from fibrosis) have increased BAL fluid and lung tissue inflammatory cells numbers compared to WT... It is also that if as the authors say "bleomycin-induced lung fibrosis depends on the preceding inflammatory response (Moeller et al., 2008)", then why is the lymphocytes accumulation in the lung parenchyma (which is supposed to correlate with fibrosis severity) in Fig 4b and Supp Fig S3 similar between gp130757F;Stat3+/- and gp130dStat mice despite these mice displaying differing levels of fibrosis (as shown in Fig 2).

We would like to point out to this reviewer that our experiments with Rag-mutant mice provide genetic evidence for a critical involvement of lymphocytes rather than innate immune cells to bleomycin-induced fibrosis in gp130(757F) mice. These cells are indeed reduced in the lung parenchyma of gp130(757F);Stat3+/- and gp130dStat mice (Figure 3b and Supplemental Figure 4b) and not, as implied by the reviewer, increased. This is particularly evident for B-cells, which based on our additional new genetic data using B-cell deficient gp130(757F);uMT mice (Supplementary Figures 4d and 4e), functionally contribute to belomycin-induced fibrosis and excessive collagen transcription in gp130(757F) mice. We now refer to this finding in the Result section on p10. On the other hand, our genetic assessment provides clear-cut evidence for a functional involvement of IL6 to bleomycin-induced fibrosis and we now provide additional data that the levels of IL6 remain below detection in gp130dStat3 mice, even after challenge with bleomycin (Supplementary Figures 3d). Our data therefore suggest that the most critical aspect of the inflammation preceding the fibrotic response is the extent of (i) IL6 production and (ii) parenchymal B-cell accumulation, both of which are low in gp130(757F);Stat3+/- and gp130dStat mice when compared to their susceptible gp130(757F) counterparts.

Move Fig 4d to Supp Fig S3.

This has been done as suggested.

In Supp Fig S4...It would be preferable to include gp130dStat mice in this Fig as presumably they don't show any activation of Stat3 from gp130 and this may yield a more concise result as opposed to partial suppression of gp130-Stat3 signal in gp130757F;Stat3+/- mice.

We now provide additional Western blot analysis (Supplementary Figures 5b) showing excessive pSmad3 levels in TGFβ-stimulated gp130dStat cells, consistent with our previous observation that excessive Stat3 activation desensitizes TGFβ-responsiveness of gp130(757F) cells (*Jenkins et al*., Nat Med 2005)

Is Osm playing a role in the gp130757F lung fibrosis phenotype? Also Osm is used to induce excessive Stat3 signalling in WT mice, yet there is no evidence provided that Stat3 activation in the lung is substantially increased in WT mice with Osm exposure.

We provide additional data that suggests that the level of Osm transcription does not change significantly in response to bleomycin challenge of wild-type or gp130(757F) mice (Supplementary Figures 2d). We also included additional data documenting an increase in pStat3 levels in lung tissue from mice exposed to the Osm adenoviral expression vector (Supplementary Figures 5e).

The statement relating to Smad2dExon3 expression that "we observed an increase in its expression between bleomycin-challenged wild-type and gp130757F mice (Figure 5d." is misleading...Remove or reword sentence...Similarly this is referred to in the Discussion, and it is unclear what data

supports the latter part of the sentence "we observed increased abundance of Smad2dExon3-specific mRNA in bleomycin-challenged gp130757F compared to gp130wt mice, which was further exacerbated in gp130757F mice in an IL6/Stat3-dependent manner". …and… *The data for Smad2dExon3 splice variant is not convincing, and the statement that "Since the formation of trancriptionally active pSmad3 is impaired in gp130757F lung fibroblasts (Supplementary Figure S4b), this result is likely to reflect the effect of increased production of the Smad2dExon3 protein" is purely speculation because no protein expression data for the splice variant is shown.*

We agree with this reviewer that our data provided on *Smad2dExon3* expression are too speculative at this stage and that therefore the clarity of the manuscript is improved by removing all the data and reference to *Smad2dExon3* expression.

Reviewer #2

1. The different mice are all compared to one time-point (30 days post bleomycin application). This limits the analysis in that the dynamic development of fibrosis is not assessed or depicted.

To provide evidence for the onset and resolution of fibrosis we have included additional data showing collagen levels in wt, gp130(757F), gp130(757F);Stat3+/- and gp130dStat mice 14 and 30 days after bleomycin challenge (Figures 2a).

2. The authors report using Bleomycin at 0.05U/kg BW. This is two potencies lower than most other users.

The reviewer has detected a typographical error in our Methods section, the bleomycin dose we used was in fact 0.05 U/mouse (i.e. 2U/kg).

3. …Protein analysis and phosphorylation of Smads should be addressed as mRNA expression levels do not correctly assess activation.

This comment relates to our transcript analysis of *Smad2dExon3* expression. In light of suggestion be by reviewer #1 we have removed all data relating to *Smad2dExon3* expression from the revised manuscript.

4. ….duplicated Figures in the submission (Fig 2, top lane is identical to Fig. 5a, top lane).

We have included additional new panels of bleomycin-treated wild-type mice as controls for the new Figure 4 (i.e. old Figure 5), new Figure 6 (i.e. old Figure 7), new Supplemental Figure 2b (i.e. old Supplemental Figure 1b), new Supplemental Figure 3a (i.e. old Supplemental Figure 2a) and new Supplemental Figure 4d (i.e. old Supplemental Figure 3d).

Reviewer #3

Some experiment methods are not adequately described. For examples, the legend for Figure 4c is not understandable (i.e. timing and logistics of bone marrow transplants).

The legend of the new Figure 3c (i.e. old Figure 4c) has been amended to aid interpretation of the data.

There is reference to embryonic fibroblasts in the methods section, but no corresponding data in the manuscript.

Data depicted in the new Figure 2c (i.e. old Figure 3c) was derived from experiments using embryonic fibroblasts, the text in the Result section on p8 and the legend have been amended to clarify this.

…Cool 2006 and Moodley 2003 do not show that epithelial injury causes the proliferation of fibroblasts and sub-epithelial myofibroblasts that are associated with excess collagen production.

We have amended the text in the Introduction section on p4 accordingly.

Have the authors performed bone marrow transplants using Stat3 heterozygous mice as donors and hosts? Similarly, the authors could address whether TGFB is sufficient to induce fibrosis in Stat3 heterozygous mice.

We have not performed transplantation experiments with bone marrow from Stat3+/- mice, because we predict that these cells would confer reduced susceptibility to bleomycin-induced fibrosis of gp130(757F) host similar to bone marrow derived from gp130dStat mice. However, a potential difference between the effect of gp130dStat and Stat3+/- bone marrow would be difficult to functionally interpret because the former mutation ablates all Stat3 signaling from gp130 while the later reduces Stat3 signaling from gp130 as well as other unrelated cytokines/growth factors that confer Stat3 activation. The focus of the present work is to document a Smad3-independent mechanism by which Stat3 promotes fibrosis rather than addressing whether Stat3 is required for TGFb-mediated fibrosis. The latter issue will be subject of future studies encompassing, among others, the experiment suggested this reviewer.

How do the authors reconcile their data with the previously published finding that transgenic overexpression of IL6 did not cause pulmonary fibrosis (Kuhn et al AJRCMB 22: 289-295 (2000)? Why is Osm sufficient to cause fibrosis but not IL6?

Our study design here is based on an injury model, with associated inflammation and regeneration, while the study by Kuhn et al explores the function of airway-specific transgenic IL6 overexpression on tissue homeostasis in the uninjured lung. We have previously observed that that IL6 family cytokines are largely dispensable for the homeostatic function of the intestinal epithelium, but are required during colitis-associated wound healing. We therefore predict that the transgenic mice described by Kuhn *et al.* might display an exaggerated fibrotic response to bleomycin-mediated injury of the lung epithelium.

Notwithstanding the difference between transgenic overexpression of IL6 from the Clara cell-specific CC10 promoter and the adenoviral expression Osm, we believe the likely reason for Osm (but not IL6) causing fibrosis of the uninjured lung (Fig 4c) may arise of differential expression of the Osm and IL6-specific receptor subunits required for gp130 activation. Indeed, it is well recognized that inflammation result in production of the soluble IL6-receptor isoform which confers responsiveness to cells lacking expression of the trans-membrane version of the IL6 receptor as a mechanisms referred to as IL6-trans signalling (Dogaci et al., 2005, J Clin Invest 115:313).

Based on previous publications form this group (e.g. Moodley et al 2003), one would expect that constitutive Mapk signaling in fibroblasts would exacerbate pulmonary fibrosis. The opposite finding is reported here (i.e. deltaStat mice were partially protected from bleomycin-induced pulmonary fibrosis.

Fibrosis is the end result of many processes including inflammation, epithelial regeneration, recruitment, proliferation and differentiation of fibroblasts into myofibroblasts, increased collagen production and reduced collagen degradation. Therefore, Mapk-mediated proliferation of cultured fibroblast does not adequately reproduce all aspect required to yield a fibrotic response in vivo. Indeed, evidence by Ramos *et al.*, (*AJRCMB 2001*) demonstrated reduced fibroblast proliferation in fibrotic lesions, suggesting that the extent of proliferation of IPF fibroblasts in vitro does not accurately predict fibrotic response of gp130 mutant mice in vivo.

Does administration of IL6 to deltaStat mice cause fibrosis?

In light of the our new additional data showing a similar extent of bleomycin-dependent IL6 induction between wt and gp130(dStat) mice (Supplementary Figures 3d), and our observation that genetic reduction of Stat3 in wt mice confers resistance to fibrosis (Figures 5a and 6b), suggests that IL6 stimulation of gp130(dStat) mice may not cause fibrosis. However, we have not formally addressed this question with an in vivo experiment.

The authors conclude from their bone marrow transplant experiments that gp130 signaling in the parenchyma is pro-fibrotic. What are the data to support this conclusion?...Moreover, in naïve wt mice (Fig4C) and wt mice (Fig4D) why aren't the levels of Collagen increased 21d after bleomycin?

In Figure 4c (now new Figure 3c) each individual histogram bar compares the extent of change to lung collagen between saline and bleomycin-treated mice of a given genotype combination of host and donor bone marrow cells, with for instance "100 percent change" indicating twice the amount of collagen in the bleomycin-treated lung compared to the saline treated lung. This

response, albeit varying between genotype combinations, was significant for all combinations, and we now clarify this finding with an asterisk above each bar. Accordingly, we conclude that the excessive fibrotic response observed in gp130(757F) hosts is mediated by excessive Stat3 signaling in bone marrow derived cells, but that the response in wild-type mice occurs independently of gp130 genotype of the hematopoietic compartment. We have now clarified the text in the Result section on p9 accordingly.

Similarly, each histogram bar in the old Figure 4d (now new Figure 3d) represents the comparison of col1I1a expression levels between saline and bleomycin-treated of the indicated genotype, where "1 fold relative change" indicates twice the amount of collagen transcripts in the bleomycin-treated compared to the saline-treated lung. Again this is now clarified by inclusion of asterisks above individual bars. In light of these clarifications, collagen levels are indeed significantly and consistently increased in (naïve) wild-type mice 21d after bleomycin challenge throughout all experiments depicted in this manuscript.

Is the histology from bleomycin treated 757F;Ragnull mice normal?

Histological assessment of lungs from bleomycin-treated 757F;Rag-/- mice shows similar fibrosis to wild-type mice. However, we observed epithelial dysplasia in the 757F;Rag-/- mice that is not evident in wt mice. We have now included additional new histology data for these mice (Supplementary Figures 4d).

The quality of the histology and micrographs is poor. The trichrome staining is variable and this makes comparison of data difficult (compare 757F saline in Figure 2 and Figure 5). Some images are used as controls in multiple experiments (Figure 2 and 5 wild type, saline and bleomycin).

We have included new micrographs comprising most of the Masson's trichrome stains to improve the quality of micrographs and standardise staining throughout the manuscript (Fig 1, Fig4a and Fig 6a as well as Supplementary Figures 2b, 3a and 4d).

If pStat3 is increased in IPF, then what does pStat3 staining look like in a normal lung?

We predict a therapeutic window for targeting of IL-6/Stat3 signaling to arise from the difference of STAT3 activity between affected and unaffected parts of the lungs of IPF patients. We therefore have not assessed pSTAT3 staining in "normal" human lung tissue which is very difficult to obtain.

No conclusions about the cell types in which pStat3 is localized can be drawn from the images in Fig 6. This would require at least double immunofluorescence ad preferably confocal microscopic analysis.

We have included new additional data comprising cytokeratin/pStat3 and CD45/pStat3 double immunofluorescence on IPF lung tissue to document significant pSTAT3 staining in non-epithelial and non-haematopoietic cells in fibrotic lung tissue (new Figure 5c). We interpret this staining as indicative for pSTAT3 activity localizing to myofibroblasts within these lesions.

2nd Editorial Decision 17 January 2012

Thank you for the submission of your revised manuscript "Genetic partitioning of interleukin-6 signalling in mice dissociates Stat3 from Smad3 mediated lung fibrosis" to EMBO Molecular Medicine and please accept my apologies for the delayed reply. We have now heard back from the referees whom we asked to evaluate your revised manuscript.

As you will see, while reviewer 3 is moderately positive about the revised study (but still raises concerns), reviewer 1 is much more reserved and still raises significant concerns regarding the unequivocal interpretation of the data. As such, the reviewers would not support publication of the revised manuscript in EMBO Molecular Medicine at this stage and thus, we feel that the level of support provided by the reviewers remains too limited. Since it is journal policy to allow a single round of major revision only, I see no choice but to return the manuscript with the message that we cannot offer to publish it.

Given the potential interest of the findings, we would, however, have no objection to consider a new

manuscript on the same topic if at some time in the near future you obtained data that would considerably strengthen the message of the study and address the referees concerns in full. To be completely clear, however, I would like to stress that if you were to send a new manuscript this would be treated as a new submission rather than a revision and would be reviewed afresh, in particular with respect to the literature and the novelty of your findings at the time of resubmission. If you decide to follow this route, please make sure you nevertheless upload a letter of response to the referees' comments.

At this stage of analysis, though, I am sorry to have to disappoint you. I nevertheless hope, that the referee comments will be helpful in your continued work in this area and I thank you for considering EMBO Molecular Medicine.

Yours sincerely,

Editor
EMBO Molecular Medicine

***** Reviewer's comments *****

Referee #1:

While the authors have somewhat adequately addressed most of concerns, it is unfortunate that some of these raised still have not been addressed satisfactorily, with examples below. Also of concern to me are some of the factual inconsistencies of some of the responses, and the revised manuscript and authors' response having numerous errors which made for difficult reading. For instances (see also examples listed below), the data presented in Figures 5a and b do not reflect the text reference to them (on pages 11 and 12), and there are no labelled Figures 7a-b despite there being text on page 13 referring to such figures.

Also, one of the fundamental mechanisms of this manuscript is that the level of Stat3 activity correlates with the extent of fibrotic disease. Therefore, in gp130(Y757F) mouse crosses on a Stat1-/- (disease same as gp130(Y757F)) and Il6-/- (disease reduced compared gp130(Y757F)) background, the authors should confirm that the level of Stat3 activity is elevated and reduced respectively. Unfortunately for the Stat1-/- cross the authors ignored this request. In response to the question "What are the Stat3 and Stat1 activation level like in the lungs of bleomycin-treated gp130757F;Il6-/- mice", they say "we were not able to generate sufficient amounts of tissue from lungs of gp130(757F);Il6-/- mice to perform this analysis." This is surprising since they are able to isolate RNA from frozen lung tissue for mouse strains used, and have also prepared lung homogenates. That lysates are apparently not available for such simple and yet key analyses regrettably reflects poorly on the experimental design.

I also note it is disappointing that the authors chose to remove all data and text related to Smad2dExon3 in response to the requests they examine the expression level of Smad2dExon3, rather than measuring the expression. This to me lowers the impact of story now they have been unable to consolidate upon the Smad3 angle of the paper.

In relation to the original comment "Fig 2: The 757F mice appear the same as those recently reported (Ruwanpura et al.)....and to develop in a spontaneous manner lung inflammation and emphysema. The authors need to address this issue which potentially confounds their conclusions. It is unclear what were the age of mice used in this study? ... it would appear that at least the 757F mice with saline shown in Fig 2 may have this lung abnormalities already (eg airspace enlargement).", on reviewing the previous manuscript published by authors I concerned about what appear to be several factual inaccuracies in their response.
Firstly, in their earlier paper (Figure 2 and text) it is clearly shown by histology and FACS that gp130(757F) mice develop inflammation by 6 months. However the authors say "we have reported that gp130(757F) mice spontaneously develop mild emphysematous changes at 6 months of age. However, at this age we could not detect inflammatory changes per se as neither BAL fluid cellularity nor histological assessment revealed increases in numbers of inflammatory cells". The

fact these mice are reported to have lung inflammation before bleomycin treatment seems therefore to have been ignored by the authors in terms of their data interpretation.
Secondly, there was no data for mean linear intercept in their previous paper that I could find, yet the authors say "In the Ruwanpura et al. paper we characterize the emphysematous changes in gp130(757F) as (i) increased lung volume; (ii) reduction in the mean linear intercept value; and (iii) the loss of alveoli.".
Thirdly, in their earlier paper their data in Table 1 clearly shows emphysema changes occurring in 1 month old mice. Since 8-12 week old mice are used in the current study, this still raises issues that the gp130(757F) already have lung alterations even before bleomycin treatment, which may confound data interpretation.
At the very least the authors should acknowledge these issues in the manuscript and include in their data interpretation.

Since a key mechanism of the paper is that increased Stat3 activation aligns with increased fibrosis, it is a pity that the authors were unable to source enough lung material to perform basic and yet key western blotting for Stat3 activation levels in their Stat1-/- and IL-6-/- crosses with gp130(Y757F) mouse. This is surprising since they could isolate RNA from frozen lung tissue for mouse strains used in study, and have prepared lung homogenates; therefore this lowers ones confidence in the experimental design. Why couldn't they use at least qRT-PCR for Stat3 genes?

The additional data documenting changes to cellularity and composition of BALF following bleomycin challenge of gp130(757F);Il6-/- mice is now in Supplementary Figure 3e, and not "Supplementary Figures 3c" as they say in their response.

In their response..."This is particularly evident for B-cells, which based on our additional new genetic data using B-cell deficient gp130(757F);uMT mice (Supplementary Figures 4d and 4e), functionally contribute to belomycin-induced fibrosis and......", I cannot find Supplementary Figure 4e in the manuscript - is this missing?

In their response..."we now provide additional data that the levels of IL6 remain below detection in gp130dStat3 mice, even after challenge with bleomycin (Supplementary Figures 3d).", I could not find IL-6 data in Supp Fig 3d? Do the authors mean Supp Fig 3c?

On page 11 of the manuscript the statement "We therefore challenged gp130757F and gp130757F;Smad3-/- mice with bleomycin and observed 21 days later in both genotypes of mice a profound fibrotic response that was characterized by excessive hydroxyproline accumulation and collagen deposition in the pulmonary interstitium (Figures 5a and 5b)" is confusing because Fig 5a and b show Massons trichrome and pStat3 immuno data from wt and 757F mice. This is also case for the statement "This was in contrast to Smad3 deficiency conferring protection from bleomycin-induced lung fibrosis (Figures 5a and 5b)" on page 12. This needs to be corrected.

On page 13 the authors refer to "Figures 7a-b", but no such figures exist. Please clarify and/or correct.

Referee #3:

The revised paper has been improved. However, there are still some points that need to be revised before the paper can be recommended for publication.

1. A major claim of the paper is that pStat3 is increased in "cells that collectively comprise the fibrotic areas" (page 12). However, Figure 5a and Fig 5b (incorrectly referred to multiple times in the text as Fig 6) are very difficult to interpret and higher magnification images are needed in combination, ideally, with markers for different stromal cell types. Are the positive cells stained brown? In Figure 5c it appears that the cytokeratin+ cells are weakly positive compared with the control.

2. More information is needed about the precise pathologic diagnosis of the 3 patients from whom

the samples in Fig 5b were obtained.

Minor points

1. The legend to Figure 5 is a mess. (c) and (b) are in the wrong order, there are no arrows shown in (c) and there are typos.
1. Page 10 delete duplicated "observed"
3. increase
4. Page 13 There is no Figure 7

Rebuttal 17 January 2012

We are naturally disappointed about your decision based on the comments made by Referees #1 and #3 upon their careful review of the resubmitted version of our manuscript.

Unfortunately, we now realize that the uploaded version of the revised manuscript was the penultimate version, and this explains the numerous inconsistencies between the text and the corresponding figures that both referees had identified. As the corresponding author I naturally have to take on full responsibility for this unfortunate mishap and the dissatisfactory response it triggered by the referees.

However, we also believe that some of the major issues raised by the two referees are factually incorrect. In particular:

- Referee #1 is incorrect with his/her statement that "..these mice are reported to have lung inflammation before bleomycin treatment.." because it is clearly stated throughout the manuscript (and acknowledged by this referee later on) that the mice used here were between 2-3 month of age, whereas the FACS analysis referred to by Referee #1 (i.e. Fig 2 in Ruwanpura et al.) was conducted with 6 month old mice.

- Referee #1 raises his/her concern about our decision to remove Smad2?Exon3 data. This referee commented previously that the sentence describing the only Smad2?Exon3 data shown in the original submission (Figure 5d) was "...misleading.." and should be "..removed or reworded." Therefore, we followed the referees request to remove this sentence and the accompanying figure. This decision also appeared logical in light of our inability to source antibodies that could unambiguously distinguish between the naÔve and delta form of Smad2 and hence confirm our original observations by protein analysis as had been requested by Referee #2.

- Referee #1 originally queried specifically about the "...activation level for Stat1 and Stat3..." in gp130(757F);IL6-KO mice. As stated in our response, we had insufficient material available from the lungs of these mice to perform the necessary protein blots to assess for the activated, tyrosine phosphorylated forms of Stat1 and Stat3. If the reviewer would have given us the opportunity in his original request to document activation level for Stat1 and Stat3 also by surrogate parameter (i.e. "...at least qRT-PCR for Stat3 genes..", as stated in his/her comments now), we could have happily included this data in our revision. Among the many collective issues originally raised by the three referees, this is the only one that we decided to not follow up experimentally, as we feel that Referee #1 raises undue concerns about the linearity of the signalling pathways between IL6, gp130 and Stat3. There is overwhelming and complementary genetic evidence for the latter in many different phyla, and we have extensively characterized reciprocal activation of Stat1 or Stat3 in our previous analysis of gp130(757F);Stat1-KO and gp130(757F);Stat3+/- (Ernst et al., JCI 2008).

- Referee #3 originally requested for the analysis of our human samples that "..This would require at least double immunofluorescence..". Accordingly, we clearly showed in the revised submission evidence for activated Stat3 in non-CD45+ (hematopoietic) and in non-cytokeratin+ (epithelial) cells around the fibrotic areas. It would seem unfair for this Referee to now raise additional issues (i.e. "..double immunofluorescence with marker for different stromal cells..", or "..a need about the

precise pathological diagnosis of the patients..") when these issues should have been raised during the initial review to give us an opportunity to address them.

Although we understand the Journal's policy about a single round of review only, we hope that on balance you might consider allowing us to submit the final revised version of our manuscript and to address the contentious issues that the two referees have raised in their most recent comments. We are also curious about the comments submitted by Referee #2.

We are looking forward to hearing from you.

Editorial Correspondence 02 February 2012

Our editor kindly asks you to please send us your final version of your manuscript and the point-by-point response, in order for her to discuss it with the referees again.

Please send the files via email to contact@embomolmed.org . Should they be too large, please send them via yousendit.com.

Regarding your question about referee # 2: Referee # 2 had not been asked to rereview your manuscript.

Thanks and best regards,

Editorial Assistant
EMBO Molecular Medicine

3rd Editorial Decision 12 March 2012

Thank you for the submission of your revised manuscript "Genetic partitioning of interleukin-6 signalling in mice dissociates Stat3 from Smad3 mediated lung fibrosis" to EMBO Molecular Medicine. I now discussed the manuscript again with the referees and I had the chance to re-read it, the reports and considered your letter.

Since we do acknowledge the potential interest of the findings, we would therefore be open to allow a second revision of the manuscript that would address the outstanding issues listed below.

- Regarding the concern of correlating increased Stat3 activation with increased fibrosis raised by Reviewer #1: Please include the RT-PCR data (or other 'surrogate' data) indicating increased Stat3 activation you mention in the revised manuscript. The Western blot you provided does not include control (non-phosphorylated) Stat3 levels and thus does currently not allow the conclusion of altered p-Stat3 levels in the mice.
- Regarding the concern about patient information raised by Reviewer #2: It is crucial to include information about the patients in the revised manuscript. This should include for example age and precise diagnosis. As far as I could see, the manuscript contains no information about the patients at all. EMBO Molecular Medicine also requires a statement that informed consent was obtained from all subjects and that the experiments conformed to the principles set out in the WMA Declaration of Helsinki [http://www.wma.net/en/30publications/10policies/b3/] and the NIH Belmont Report [http://ohsr.od.nih.gov/guidelines/belmont.html]. Additionally, authors must identify the institutional committee that approved the experiments.
- Reviewer #2 initially raised concerns about the quality of the images, which were not addressed in the revised version of the manuscript. Please provide not just larger pictures but higher magnification images of Fig 5A and B. In addition, to support the notion that pStat3 is increased in IPF, it is crucial to include staining of control tissue. Please also include the H&E-stained section you mentioned in the letter.

On a more editorial note, please address the following concerns:

- Please provide a Table of Contents as the first page of the Supporting Information file and provide the supporting figure legend on the same page of the supporting figure where possible.
- Please adjust the format of The Paper Explained to journal standard. You can find examples in published EMBO Molecular Medicine papers.
- Please reduce the size of the panels again so that all panels of one figure fit on one page. Please also reduce the size of the panels again that you increased during revision (for example Fig 1).
- In Fig 2A, please remove the information 'open' and 'closed' from the panel. This information is already contained in the boxes.
- Please see our Author Guidelines for Statistical Analysis of the data (http://onlinelibrary.wiley.com/journal/10.1002/(ISSN)1757-4684/homepage/ForAuthors.html#data2).

Revised manuscripts should be submitted within three months of a request for revision; they will otherwise be treated as new submissions, unless arranged differently with the editor.

I look forward to seeing a revised form of your manuscript as soon as possible.

Yours sincerely,

Editor
EMBO Molecular Medicine

2nd Revision - Authors' Response 16 April 2012

Please find enclosed the revised version of our manuscript entitled: "**Genetic partitioning of interleukin-6 signalling in mice dissociates Stat3 from Smad3 mediated lung fibrosis**" by Robert O'Donoghue and colleagues for your consideration for publication in *EMBO Molecular Medicine*.

We have addressed the three major issues that you have raised with us in your email of March 12:

(1) *"Please include the RT-PCR data (or other 'surrogate' data) indicating increased Stat3 activation you mention in the revised manuscript".* This comment relates to the issued raised earlier by referee #1 wanting to know that excessive Stat3 activity in gp130757F mice was due to IL6.

We now confirm by genetic means in a new Panel as Fig 5A and using the surrogate marker for Stat3-activity (i.e. *Socs3* expression) that sustained Stat3 activity in gp130757F mice is indeed dependent on IL6.

(2) *"It is crucial to include information about the patients in the revised manuscript".*

The requested patient information is now contained in the Materials section on p20 in addition to a table included in the Supplementary data file.

(3) *"Please provide not just larger pictures but higher magnification images of Fig 5A and B".*

We have comprehensively updated these panels and included one additional panel now displayed as Panels B-D in the Figure 5. We also have extensively rewritten the text describing these results on p12/13 of the revised manuscript.

You also requested that we:

(1) "provide a Table of Contents as the first page of the Supporting Information file".

This information is now provided and precedes all supporting information.

(2) "adjust the format of The Paper Explained to journal standard"

This section of the manuscript has been brought in to line with the Journal's requested format.

(3) "reduce the size of the panels again so that all panels of one figure fit on one page"

All revised individual figures now fit on one A4 page.

(4) "In Fig 2A, please remove the information 'open' and 'closed' from the panel"

The figure has been changed as requested.

We trust that you find that we have addressed the remaining outstanding issues to your satisfaction and are looking forward to hearing from you at your earliest convenience.
