# Supplementary material for: Genetic partitioning of interleukin-6 signalling in mice dissociates Stat3 from Smad3-mediated lung fibrosis
Source: EMBO Mol Med. 2012 Jun 8;4(9):939–51. doi: 10.1002/emmm.201100604 (PMC3491826; doi:10.1002/emmm.201100604)
Supplement: Supplementary file 2 [file emmm0004-0939-SD2.pdf]

Genetic partitioning of gp130 signalling in mice dissociates Stat3 from Smad3-mediated lung fibrosis

[1-4]Robert J. J. O'Donoghue, [5]Darryl A. Knight, [6]Carl D. Richards, [2,3]Cecilia M. Prêle,[2,3]Hui Ling Lau, [1]Andrew G. Jarnicki, [4]Jessica Jones, [4]Steven Bozinovski, [4]Ross Vlahos, [1]Stefan Thiem, [7]Brent S. McKenzie, [8]Bo Wang, [9]Philip Stumbles, [10]Geoffrey J. Laurent, [10]Robin J. McAnulty, [11]Stefan Rose-John, [8]Hong Jian Zhu, [4]Gary P. Anderson, [1,13]Matthias R. Ernst, [2,3,12,13]Steven E. Mutsaers.

Online Data Supplement

Table Of Contents

Supplementary Figure S1 .... **Page 2**

Supplementary Figure S2 .... **Page 3**

Supplementary Figure S3 .... **page 4**

Supplementary Figure S4 .... **Page 5**

Supplementary Figure S5 .... **Page 6**

Supplementary Figure S6 .... **Page 7**

Supplementary Table T1 .... **Page 8**

Supplementary Methods .... **Page 9**

Supplementary References .... **Page 10**

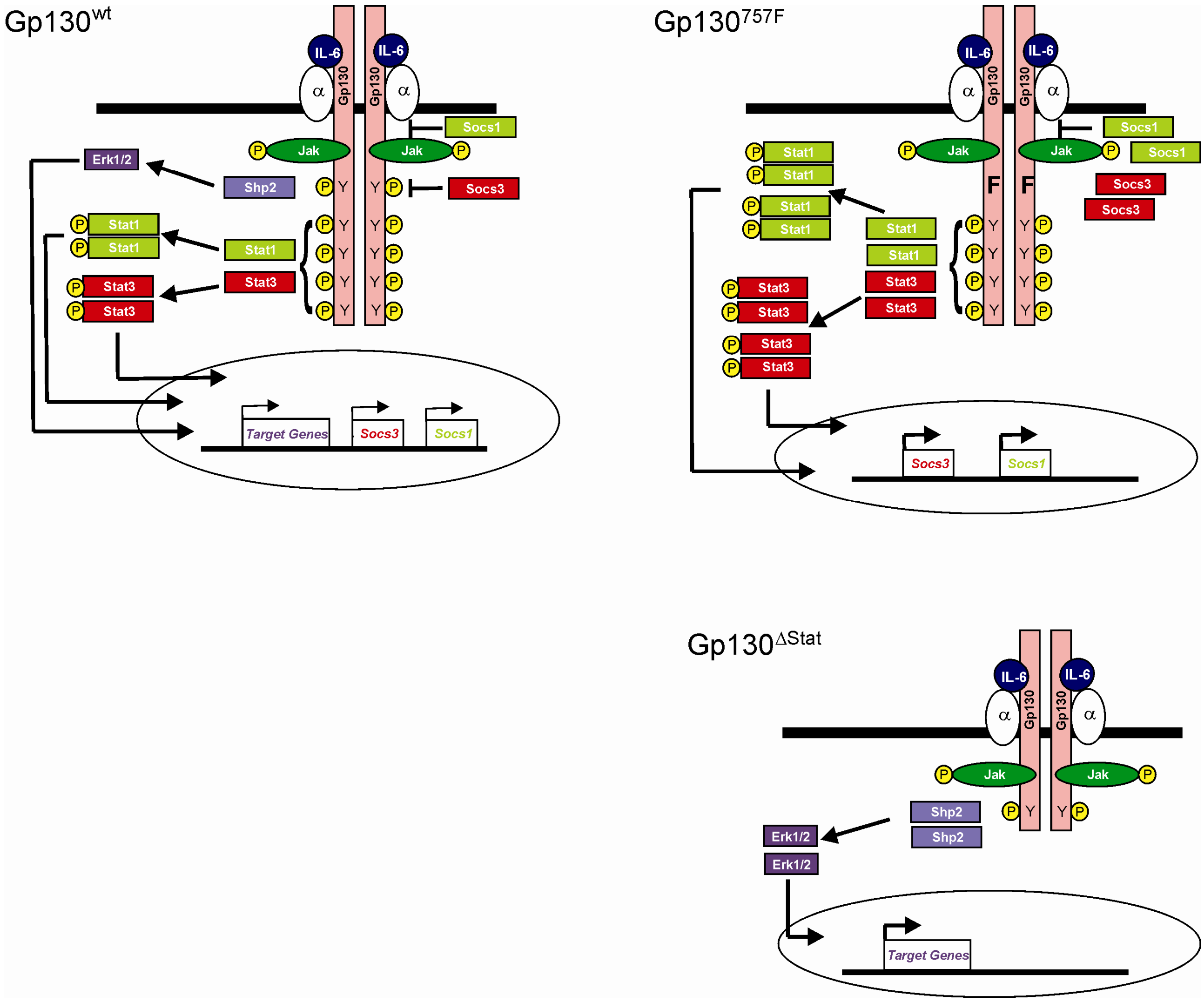

Supplementary Figure S1. Effects of Gp130 mutations on downstream signalling and gene transcription. Schematic representations of downstream intracellular signalling and gene transcription in wild-type (*gp130*^{wt}, left panel), *gp130*^{757F} (right panel) and *gp130*$^{\Delta Stat}$ (bottom panel) mice upon ligand-dependent activation of the cell surface receptor complex comprising of the IL-6 ligand, α-receptor (α) and gp130. In *gp130*^{757F} mice Shp2-Erk1/2 signalling and Socs3-mediated negative feedback are ablated by a tyrosine (Y) to phenylalanine substitution (F) at position 757 and culminating in ligand-dependent hyperactivation of Stat1/3. In *gp130*$^{\Delta Stat}$ mice Stat1/3 signalling is ablated by a truncation mutation located distal to the tyrosine 757 position culminating in ligand-dependent excessive Shp2-Erk1/2 signalling.

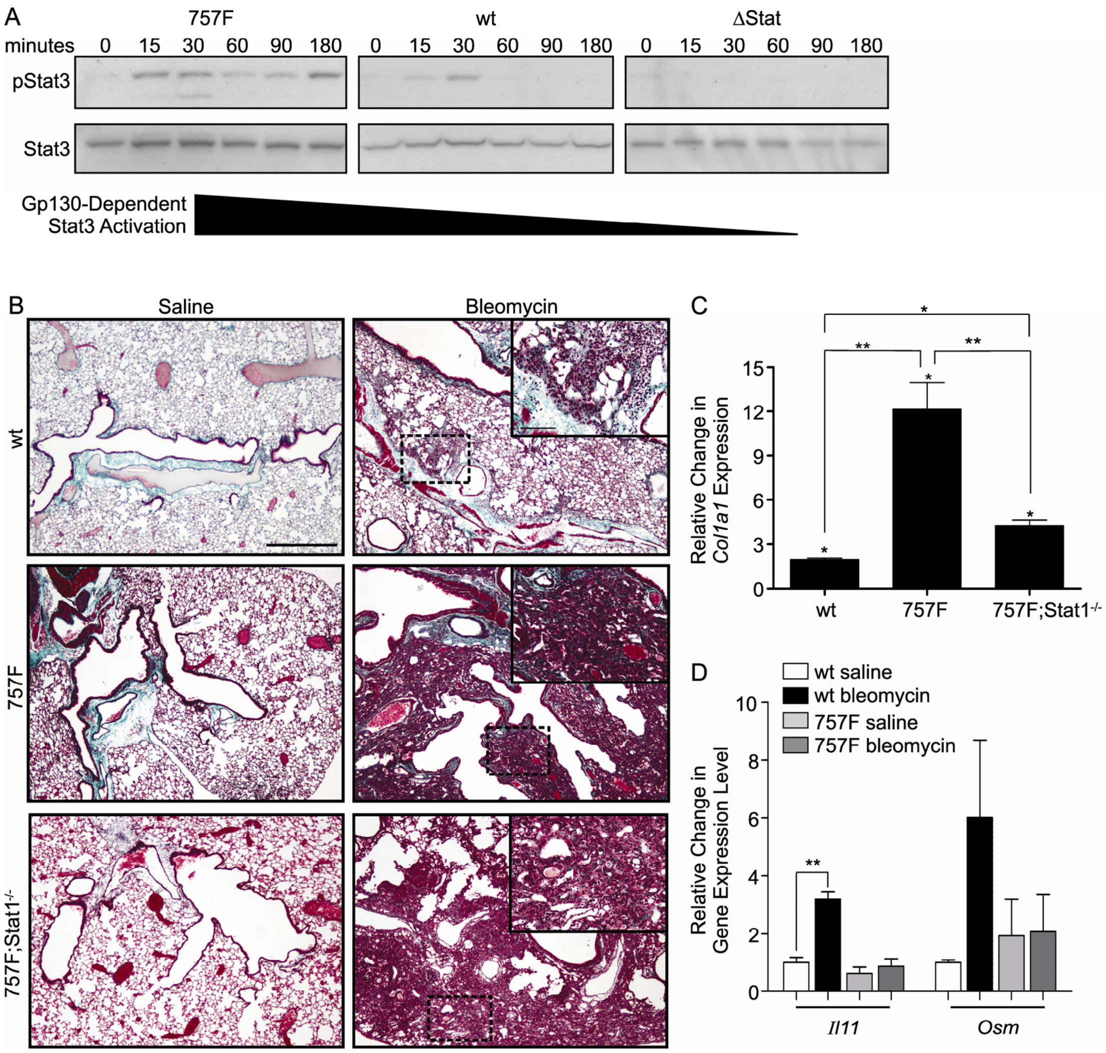

Supplementary Figure S2. Molecular contribution of Stat signalling to bleomycin-induced fibrosis in *gp130^{757F}* mice. (A) Stat3 phosphorylation (pStat3) in cultured lung fibroblasts derived from *gp130^{wt}* (wt), *gp130^{757F}* (757F) or *gp130$^{\Delta Stat}$* (ΔStat) mice after the indicated period of time following exposure to IL-6. Blots were reprobed for Stat3 to assess for protein loading. Blots are representative of two experiments. **(B)** Masson's trichrome stained sections of lung tissue from wt, 757F and compound *gp130^{757F};Stat1$^{-/-}$* (757F;Stat1$^{-/-}$) mice 30 days after bleomycin challenge. Images are representative of two mice. Scale bar = 500μm (insets =50μm). **(C)** qPCR analysis of *Col1a1* expression in lung homogenates 30 days after bleomycin treatment and normalized to *18s* expression. **(D)** qPCR analysis of *Il11* and *Osm* expression in lung homogenates from mice three days after bleomycin challenge and saline control mice. Data are expressed as the mean ± SEM, with * p<0.05, ** p<0.01 and *** p<0.001 by Bonferroni multiple comparisons test. Data are shown as relative to normalized values from lung homogenates of saline challenged syngeneic mice.

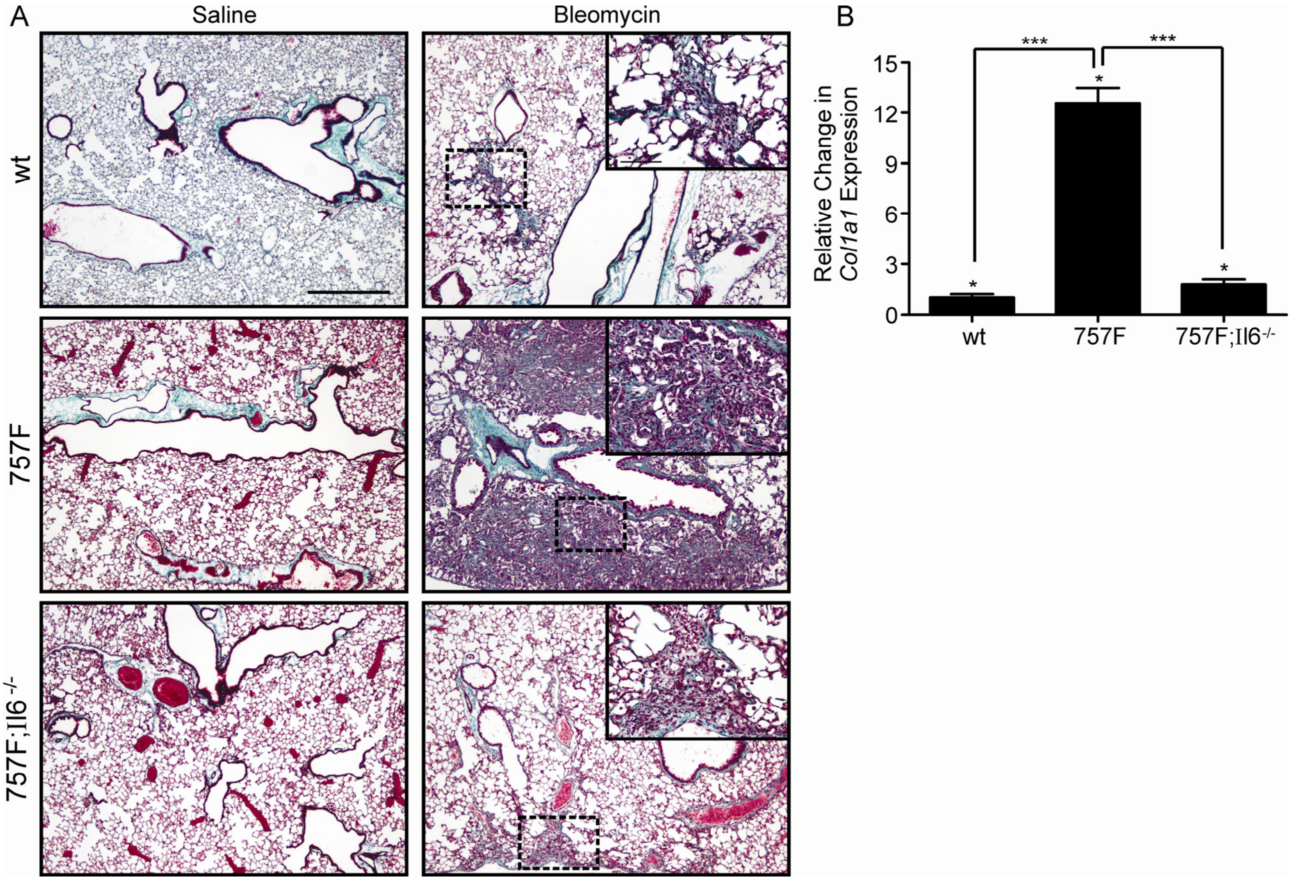

Supplementary Figure S3. IL-6 promotes pulmonary fibrosis in bleomycin challenged *gp130*^{757F} mice. **(A)** Masson's trichrome stained section of lungs from wild-type (wt), *gp130*^{757F} (757F) and compound *gp130*^{757F}*;Il6*$^{-/-}$ (757F;Il6$^{-/-}$) mice 30 days after exposure to trans-nasally applied bleomycin or saline vehicle. Images are representative of 3 mice. Scale bar = 500µm (insets = 100µm). **(B)** qPCR analysis of *Col1a1* mRNA expression in lung homogenates from wt, 757F and compound 757F;Il6$^{-/-}$ mice 30 days after bleomycin treatment and normalized to *18S* expression. *Col1a1* mRNA expression was normalised to *18S* levels and expressed relative to normalised values from lung homogenates of saline challenged syngeneic mice. Data are expressed as the mean $\pm$ SEM, with *p<0.05, **p<0.01 and ***p<0.001 by Bonferroni multiple comparisons test.

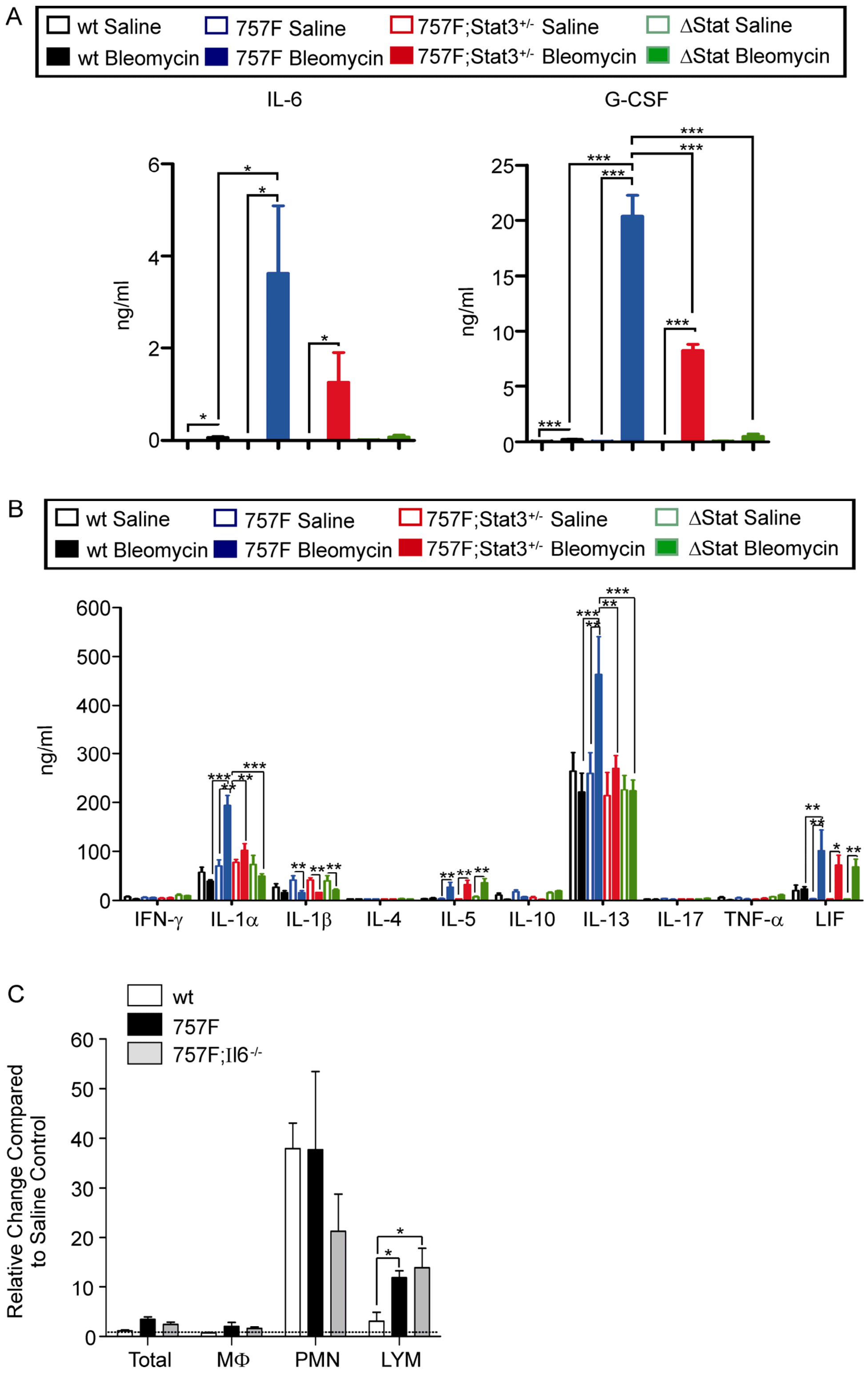

Supplementary Figure S4. IL-6 is increased in the lungs of bleomycin challenged *gp130*^{757F} mice. (A, B) BAL fluid was collected from mice of the indicated genotypes three days after exposure to trans-nasally applied bleomycin or saline vehicle and cytokine levels determined by paired capture and detection antibodies. n=3 mice. **(C)** Changes to cell numbers in BAL fluid of wt, 757F and 757F;Il6$^{-/-}$ mice 3 days after bleomycin administration relative to saline-treated mice of the same genotype. n≥4 mice. Bars indicate mean ± SEM, with *p<0.05, **p<0.01 and ***p<0.001 by Bonferroni multiple comparisons test.

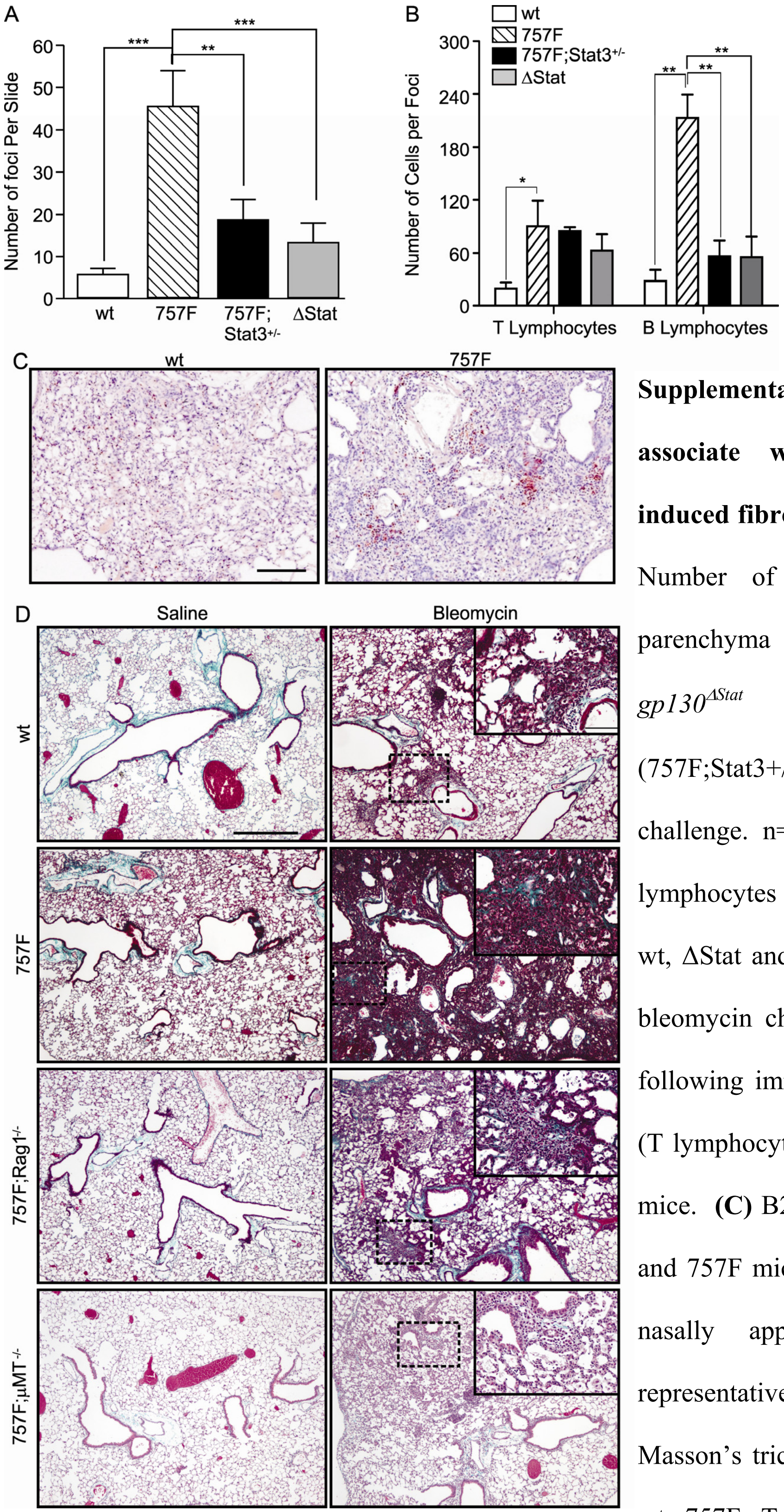

Supplementary Figure S5. Lymphocytes associate with and determine bleomycin-induced fibrotic response in *gp130*^{757F} mice. (A) Number of inflammatory cell foci in lung parenchyma of *gp130*^{wt} (wt), *gp130*^{757F} (757F), *gp130*$^{\Delta Stat}$ (ΔStat) and *gp130*^{757F}*;Stat3*$^{+/-}$ (757F;Stat3+/-) mice three days after bleomycin challenge. n=4 mice. **(B)** Number of T and B lymphocytes in foci in lung parenchyma of 757F, wt, ΔStat and 757F;Stat3+/- mice three days after bleomycin challenge. Cell types were identified following immunohistochemical staining for CD3 (T lymphocytes) and B220 (B lymphocytes). n=4 mice. **(C)** B220 stained sections of lungs from wt and 757F mice three days after exposure to trans-nasally applied bleomycin. Images are representative of 3 mice. Scale bar =500µm. **(D)** Masson's trichrome stained section of lungs from wt, 757F, T and B lymphocyte double deficient *gp130*^{757F}*;Rag1*$^{-/-}$ (757F;Rag1$^{-/-}$) and B lymphocyte deficient *gp130*^{757F}*;µMT*$^{-/-}$ (757F;µMT$^{-/-}$) mice 30 days after exposure to trans-nasally applied bleomycin or saline vehicle. Images are representative of 3 mice. Scale bar = 500µm (insets = 100µm). Data are expressed as the mean $\pm$ SEM, with * $p<0.05$, ** $p<0.01$, *** $p<0.001$ by Bonferroni multiple comparisons test on absolute data.

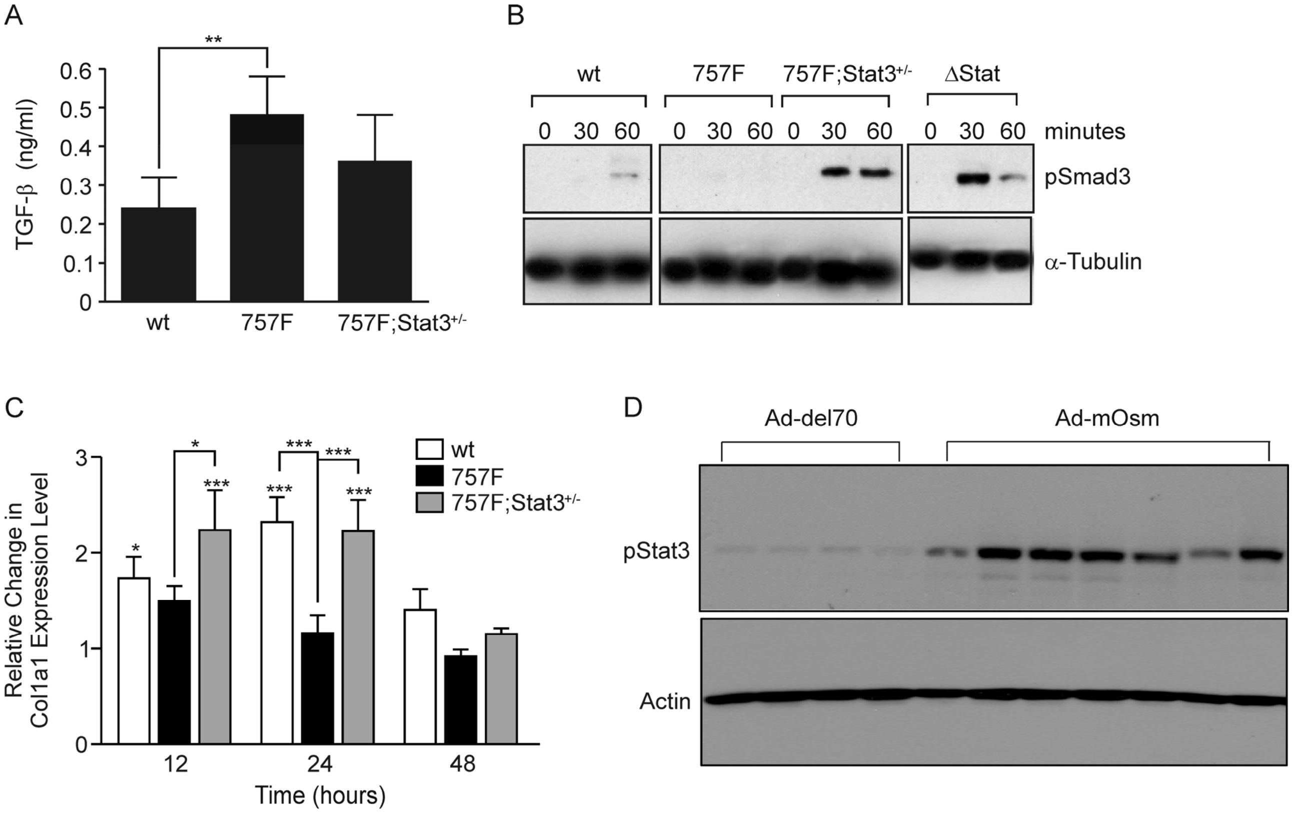

Supplementary Figure S6. Discordance between TGF-β signalling and gp130-mediated lung fibrosis. **(A)** Bioactive TGF-β was measured in serum of naïve $gp130^{wt}$ (wt), $gp130^{757F}$ (757F) or $gp130^{757F};Stat3^{+/-}$ (757F;Stat3$^{+/-}$) mice as determined by p(CAGA)$_{12}$-Luc TGF-β-reporter activity and quantified by recombinant protein added to the culture medium of transfected NIH3T3 cells. n=4 mice. **(B)** Smad3 phosphorylation (pSmad3) in cultured lung fibroblasts derived from wt, 757F, 757F;Stat3$^{+/-}$ or $gp130^{\Delta Stat}$ (ΔStat) mice after the indicated period of time following exposure to TGF-β1 (5ng/ml). Blots were reprobed for α-Tubulin to assess for protein loading. Blots are representative of two experiments. **(C)** qPCR analysis of *Col1a1* expression in cultured lung fibroblasts from wt, 757F and 757F;Stat3$^{+/-}$ mice by qPCR and stimulated in vitro with TGF-β1 (5ng/ml) for the indicated time. *Col1a1* mRNA levels are expressed as a relative change of *Col1a1*/*18S* ratios of untreated cells of the same genotype. n = 3 mice. **(D)** Stat3 phosphorylation (pStat3) in lung homogenates from wt mice 14 days after trans-nasal delivery of control adenoviral vector (Ad-del70, left) or Oncostatin M expressing adenoviral vector (Ad-mOsm, right). Each lane represents protein derived from the lungs of an individual mouse. Data are expressed as mean ± SEM, with * p<0.05, ** p<0.01, *** p<0.001 using Bonferroni multiple comparisons test.

Diagnosis	Number		Age (years)	
	Male	Female	Range	Mean
UIP	4	0	59 - 69	63

Supplementary Table T1. IPF patient information. Human tissues used in this study were retrospective paraffin embedded tissue samples taken for diagnostic purposes at Sir Charles Gairdner Hospital, Nedlands, Western Australia, Australia. All patients had a pathologist confirmed diagnosis of usual interstitial pneumonia (UIP).

SUPPLEMENTAL METHODS

For cytokine measurements, BAL fluid was collected three days after exposure to trans-nasally applied bleomycin or saline vehicle and analyzed by paired capture and detection antibodies (Milliplex, Millipore, Massachusetts, USA) for the indicated mouse cytokines.

We prepared protein lysates from cultured lung fibroblasts after stimulation with cytokine for Western blot analysis using enhanced chemiluminescence (Amersham Pharmacia Biotech) as described (Ernst et al., 2008; Jenkins et al., 2005).

We determined TGF-β-mediated transcriptional response of the *Col1a1* gene in cultured cells by qRT-PCR using mouse Collagen αI type I TaqMan® Gene Expression Assay (FAM primer/probe), eukaryotic *18S* rRNA endogenous control (VIC/MGB probe), and TaqMan® Gene Expression Master Mix (Applied Biosystems, Foster City, CA).

We detected bioactive TGF-β in whole serum using an NIH3T3 cell line expressing a Smad3 luciferase gene reporter plasmid, $p(CAGA)_{12}$-Luc, and a dual-luciferase reporter assay (Promega, USA) and recombinant TGF-β1 to construct a reference curve (Tsantikos et al., 2009).

SUPPLEMENTAL REFERENCES

Ernst, M., M. Najdovska, D. Grail, T. Lundgren-May, M. Buchert, H. Tye, V.B. Matthews, J. Armes, P.S. Bhathal, N.R. Hughes, E.G. Marcusson, J.G. Karras, S. Na, J.D. Sedgwick, P.J. Hertzog, and B.J. Jenkins. 2008. STAT3 and STAT1 mediate IL-11-dependent and inflammation-associated gastric tumorigenesis in gp130 receptor mutant mice. *The Journal of clinical investigation* 118:1727-1738.

Jenkins, B.J., D. Grail, T. Nheu, M. Najdovska, B. Wang, P. Waring, M. Inglese, R.M. McLoughlin, S.A. Jones, N. Topley, H. Baumann, L.M. Judd, A.S. Giraud, A. Boussioutas, H.J. Zhu, and M. Ernst. 2005. Hyperactivation of Stat3 in gp130 mutant mice promotes gastric hyperproliferation and desensitizes TGF-beta signaling. *Nat Med* 11:845-852.

Tsantikos, E., C. Quilici, K.W. Harder, B. Wang, H.J. Zhu, G.P. Anderson, D.M. Tarlinton, and M.L. Hibbs. 2009. Perturbation of the CD4 T cell compartment and expansion of regulatory T cells in autoimmune-prone Lyn-deficient mice. *J Immunol* 183:2484-2494.
